# Supplementary material for: Potential Tumor Suppressor Role of Polo-like Kinase 5 in Cancer
Source: Cancers (Basel). 2023 Nov 17;15(22):5457. doi: 10.3390/cancers15225457 (PMC10669931; doi:10.3390/cancers15225457)
Supplement: Supplementary file 1 [file cancers-15-05457-s001.zip › cancers-2707693-supplementary.pdf]

Supplementary Materials for:

Potential tumor suppressor role of Polo-Like Kinase 5 in cancer

Shengqin Su[1,a,+], Mary Ann Ndiaye[1,a], Glorimar Guzmán-Pérez[1], Rebecca Michael Baus[2], Wei Huang[2], Manish Suresh Patankar[3,4], Nihal Ahmad[1,4,*]

Affiliation of authors:

[1] Department of Dermatology, University of Wisconsin, Madison, Wisconsin, 53705, USA

[2] Department of Pathology and Laboratory Medicine, University of Wisconsin, Madison, Wisconsin, 53705, USA

[3] Department of Obstetrics and Gynecology, University of Wisconsin, Madison, Wisconsin, 53792, USA

[4] William S. Middleton VA Medical Center, Madison, Wisconsin, 53705, USA

[a] These authors contributed equally to this work

*__Correspondence to__:

Nihal Ahmad, Ph.D., Department of Dermatology, University of Wisconsin,

1111 Highland Avenue, Room 7045, Madison, Wisconsin, 53705

Phone: (608) 263-2532; Fax: (608) 263-5223; E-mail: nahmad@dermatology.wisc.edu

Keywords: PLK5, Cancer, Polo-Like Kinases, Tumor Suppressor

[+] New affiliation for Shengqin Su (changed after experiments were completed): Department of Radiation Oncology, Stanford Cancer Institute and Stanford University School of Medicine, Stanford, CA, USA

Supplementary Figure S1.

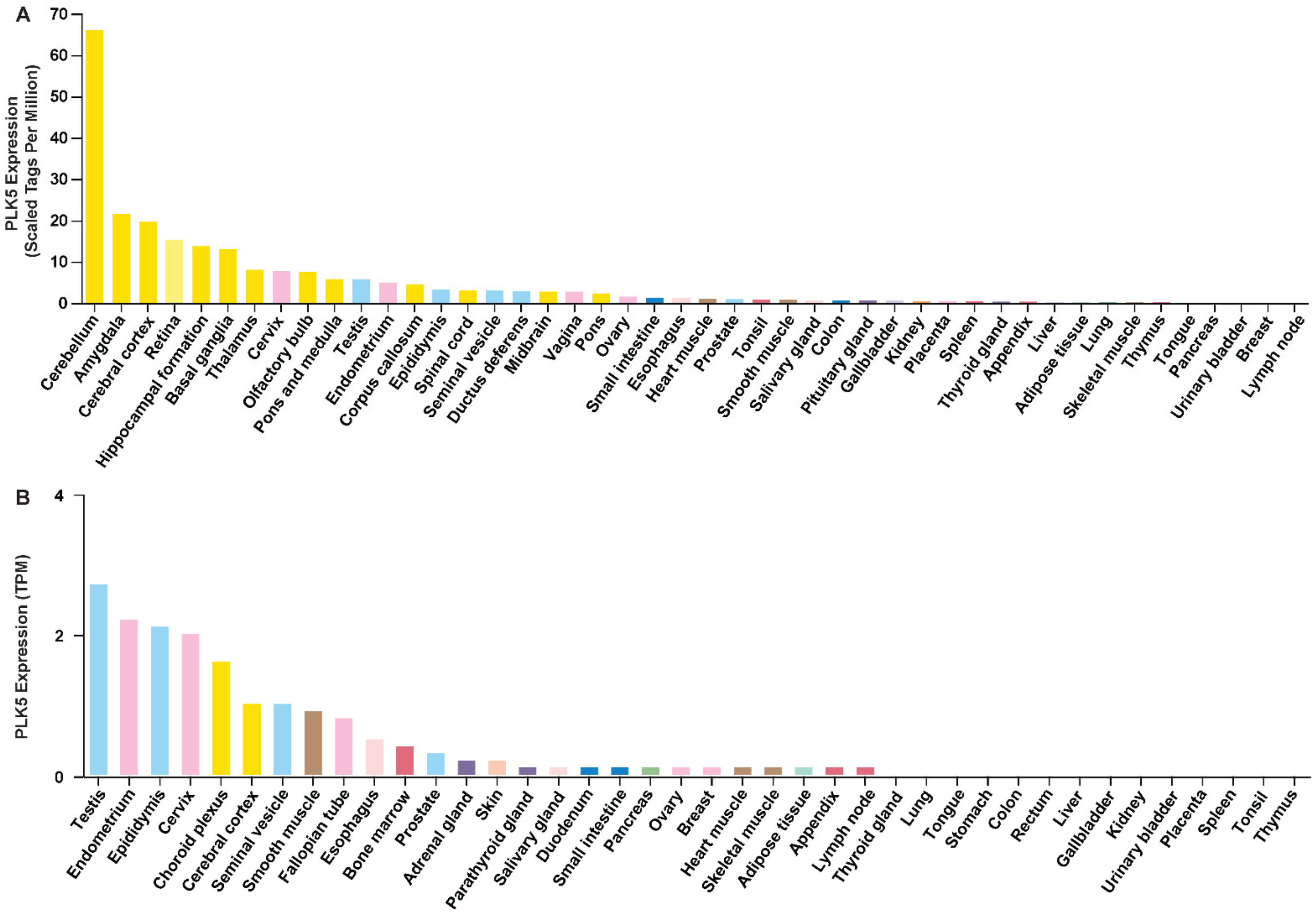

Expression profile of PLK5 across multiple tissue types. PLK5 expression profile was assessed using publicly available HPA and FANTOM5 databases. A bioinformatics analysis of RNA-Seq data was conducted to obtain the *PLK5* expression in multiple tissue types. Data from (A) HPA and (B) FANTOM5 databases are shown. Image credits for Human Protein Atlas, www.proteinatlas.org19. Images have been edited for style only and original image is available at the following URL: v23.proteinatlas.org/ENSG00000185988-PLK5/tissue.

Supplementary Figure S2.

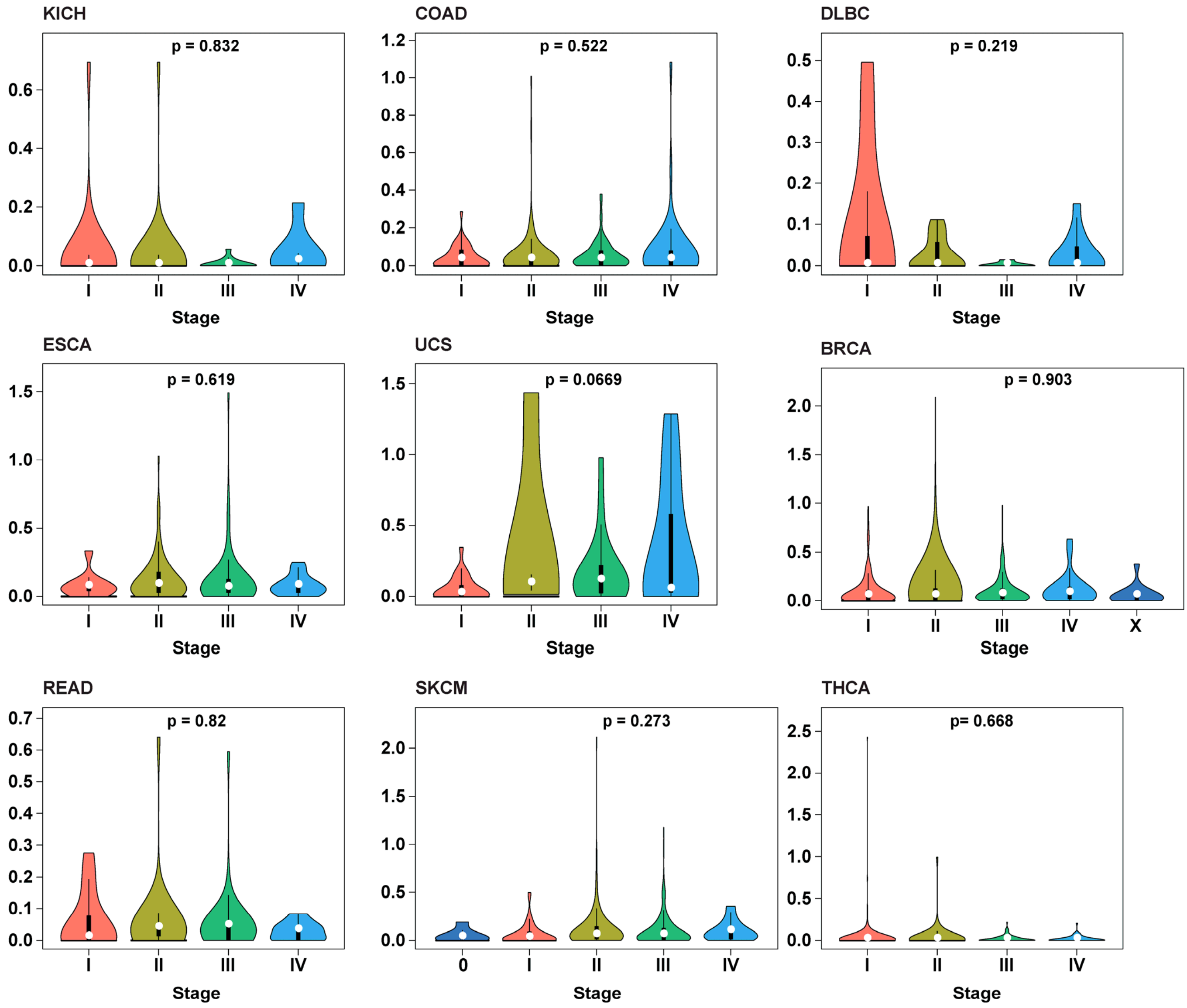

PLK5 stage-wise expression in TCGA. Stage-wise analysis was performed in tumor types available on TCGA database other than those tested via TMA. BRCA: Breast invasive carcinoma; COAD: Colon adenocarcinoma; DLBC: Lymphoid Neoplasm Diffuse Large B-cell Lymphoma; ESCA: Esophageal carcinoma; KICH: Kidney Chromophobe; READ: Rectum adenocarcinoma; SKCM: Skin Cutaneous Melanoma; THCA: Thyroid carcinoma; UCS: Uterine Carcinosarcoma.

Supplementary Figure S3.

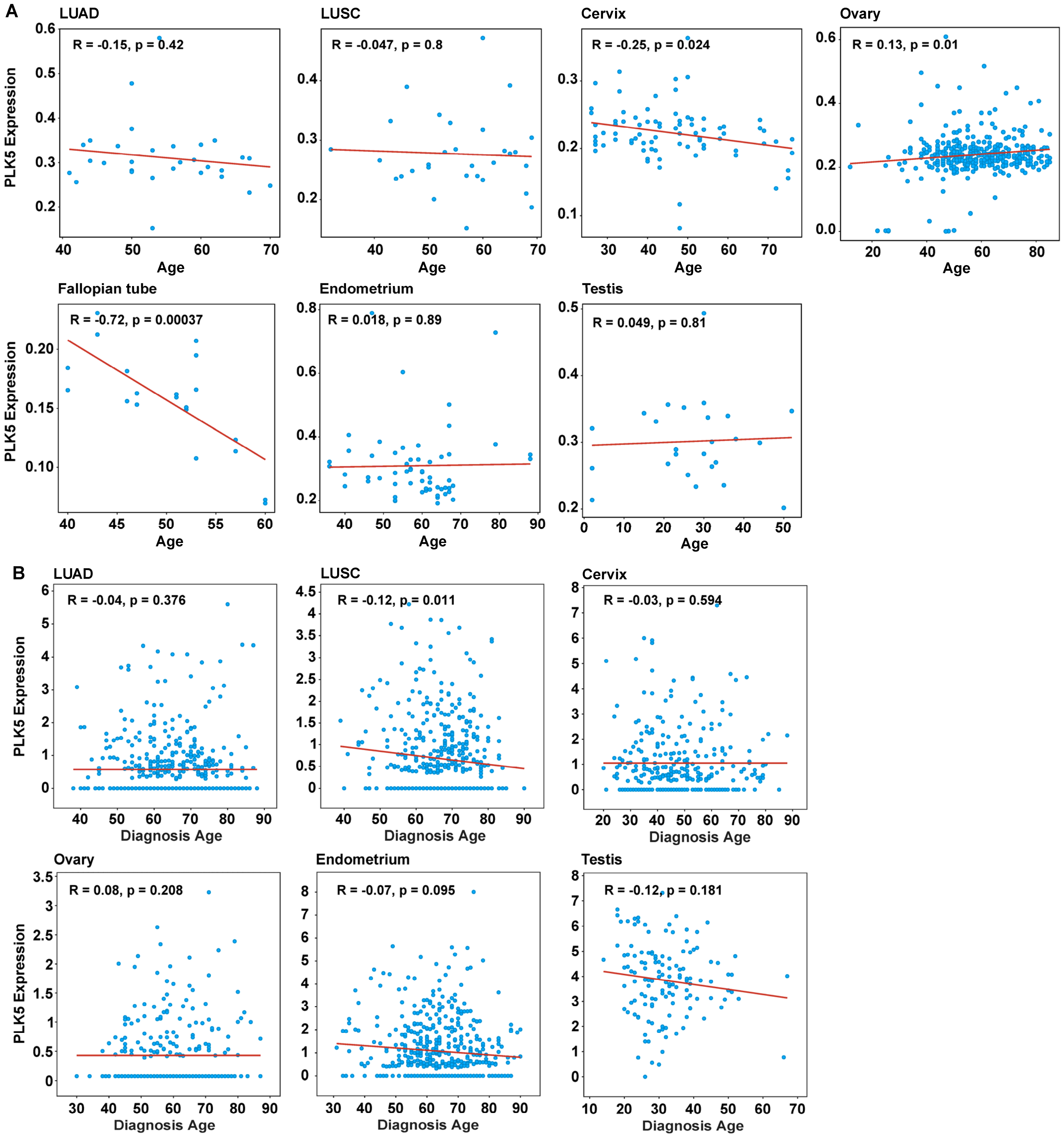

Correlation of PLK5 expression with age in selcted tumor types. A) Correlation of PLK5 protein levels with age in our TMA analysis is shown; (B) Correlation of PLK5 RNA expression with age using the TCGA database is shown. LUAD: Lung adenocarcinoma; LUSC: Lung squamous cell carcinoma.

Supplementary Figure S4.

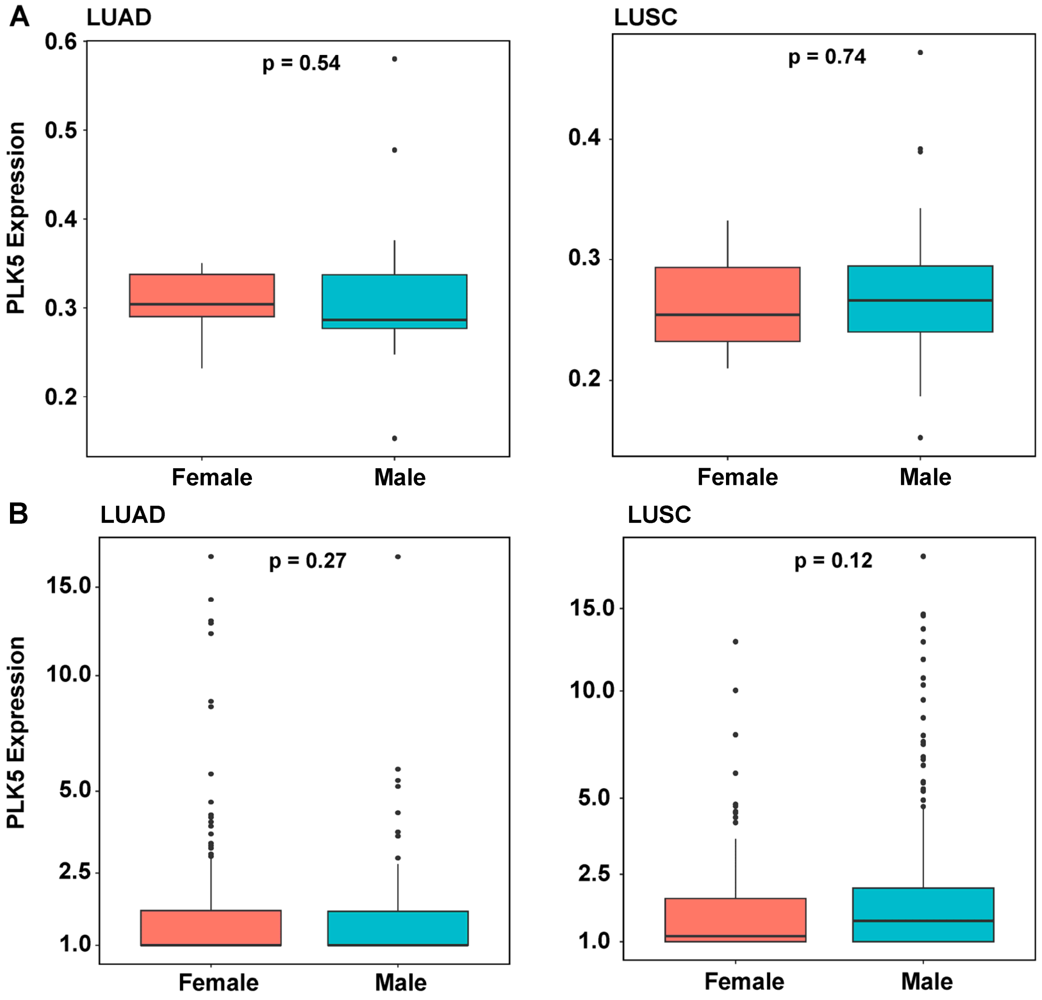

Correlation of PLK5 expression with biological sex in selected non-reproductive tissues. A) Correlation of PLK5 protein levels with sex in LUAD (lung adenocarcinoma) and LUSC (lung squamous cell carcinoma) in our TMA analysis, is shown; B) Correlation of PLK5 RNA expression in LUAD and LUSC using TCGA data is shown.

Supplementary Figure S5.

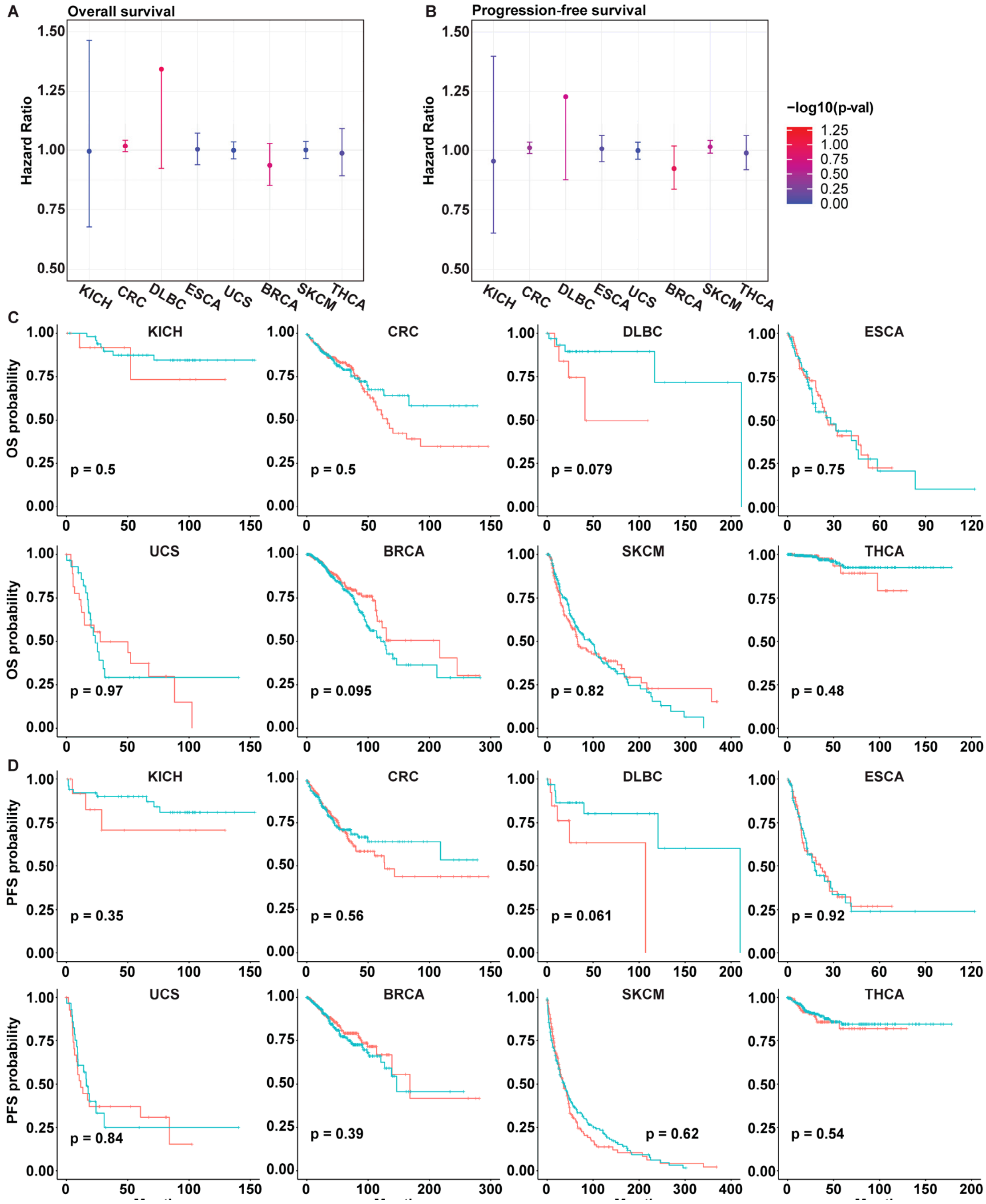

Analysis of PLK5 expression and patient survival in additional cancer types. A: Hazard ratio plot on how PLK5 expression affects overall survival in the cancer types. The error bar denotes 95% confidence interval. B: Hazard ratio plot on how PLK5 expression affects progression-free survival in the cancer types. The error bar denotes 95% confidence interval. C: Kaplan-Meier plots comparing the overall survival between PLK5-High and PLK5-Low patients. D: Kaplan-Meier plots comparing the progression-free survival between PLK5-High and PLK5-Low patients. In C and D, the red color denotes PLK5-High patients; and the green color denotes PLK5-Low patients. KICH: Kidney Chromophobe; CRC: Colorectal Cancer (combined COAD: Colon adenocarcinoma and READ: Rectum adenocarcinoma); DLBC: Lymphoid Neoplasm Diffuse Large B-cell Lymphoma; ESCA: Esophageal carcinoma; UCS: Uterine Carcinosarcoma; BRCA: Breast invasive carcinoma; SKCM: Skin Cutaneous Melanoma; THCA: Thyroid carcinoma.

Supplementary Table S1: Tissue microarray (TMA) Details

Tissue Type	Number of Cases		Cores Per Case	Vendor	Catalog Number
	Non-Malignant	Malignant			
Cervix	10	40	2	US Biomax	CR1001b
Endometrium	31	31	1	US Biomax	OD-CT-RpUTR03-02
Fallopian Tube	40	20	1	US Biomax	UTE601
Lung	25	75	1	US Biomax	BC04002a
Ovary (total)	50	417			
Normal Ovary	30	0	2	US Biomax	OV806
Ovarian Disease	20	80	1	US Biomax	OV1005b
Ovarian Cancer	0	337	1	UW BioBank	N/A
Testis	25	55	1	US Biomax	TE803

Supplementary Table S2: Clinical information of TMA samples and quantification of PLK5

	Tissue type	Sex	Age	Organ	Pathology	Stage	Grade	TNM	Position	PLK5 expression
Cervix tissues (catalog no. CR1001b)										
	Malignant	F	42	Cervix	Adenocarcinoma	IB	1	T1bN0M0	A1	0.2779
	Malignant	F	42	Cervix	Adenocarcinoma	IB	1	T1bN0M0	A2	0.2400
	Malignant	F	75	Cervix	Adenocarcinoma	I	1	T1N0M0	A3	0.1671
	Malignant	F	75	Cervix	Adenocarcinoma	I	1	T1N0M0	A4	0.1556
	Malignant	F	52	Cervix	Adenocarcinoma	IB2	1--2	T1b2N0M0	A5	0.2227
	Malignant	F	52	Cervix	Adenocarcinoma	IB2	1--2	T1b2N0M0	A6	0.1895
	Malignant	F	48	Cervix	Adenocarcinoma	I	1--2	T1N0M0	A7	0.0816
	Malignant	F	48	Cervix	Adenocarcinoma	I	1--2	T1N0M0	A8	0.1168
	Malignant	F	43	Cervix	Adenocarcinoma	IB	2	T1bN0M0	A9	0.1714
	Malignant	F	43	Cervix	Adenocarcinoma	IB	2	T1bN0M0	A10	0.1837
	Malignant	F	51	Cervix	Adenocarcinoma	IA	2	T1aN0M0	B1	0.2435
	Malignant	F	51	Cervix	Adenocarcinoma	IA	2	T1aN0M0	B2	0.2355
	Malignant	F	41	Cervix	Mucinous adenocarcinoma	IB	2	T1bN0M0	B3	0.1991
	Malignant	F	41	Cervix	Mucinous adenocarcinoma	IB	2	T1bN0M0	B4	0.2095
	Malignant	F	32	Cervix	Adenocarcinoma	IA	2	T1aN0M0	B5	0.2218
	Malignant	F	32	Cervix	Adenocarcinoma	IA	2	T1aN0M0	B6	0.2130
	Malignant	F	72	Cervix	Adenocarcinoma	IIB	2	T2bN0M0	B7	0.2102
	Malignant	F	72	Cervix	Adenocarcinoma	IIB	2	T2bN0M0	B8	0.1401
	Malignant	F	40	Cervix	Adenocarcinoma	I	2	T1N0M0	B9	0.1843
	Malignant	F	40	Cervix	Adenocarcinoma	I	2	T1N0M0	B10	0.1807
	Malignant	F	50	Cervix	Adenocarcinoma	IIIB	2--3	T1N1M0	C1	0.2461
	Malignant	F	50	Cervix	Adenocarcinoma	IIIB	2--3	T1N1M0	C2	0.2025
	Malignant	F	29	Cervix	Adenocarcinoma	II	2--3	T2N0M0	C3	0.2046
	Malignant	F	29	Cervix	Adenocarcinoma	II	2--3	T2N0M0	C4	0.2229
	Malignant	F	34	Cervix	Adenocarcinoma	IIB	2--3	T2bN0M0	C5	0.2538
	Malignant	F	34	Cervix	Adenocarcinoma	IIB	2--3	T2bN0M0	C6	0.2398
	Malignant	F	26	Cervix	Endometrioid adenocarcinoma	I	3	T1N0M0	C7	0.2592
	Malignant	F	26	Cervix	Endometrioid adenocarcinoma	I	3	T1N0M0	C8	0.2527
	Malignant	F	59	Cervix	Adenocarcinoma	IB1	3	T1b1N0M0	C9	0.2221
	Malignant	F	59	Cervix	Adenocarcinoma	IB1	3	T1b1N0M0	C10	0.2324
	Malignant	F	54	Cervix	Adenosquamous carcinoma	IB	-	T1bN0M0	D1	0.2442
	Malignant	F	54	Cervix	Adenosquamous carcinoma	IB	-	T1bN0M0	D2	0.2001
	Malignant	F	38	Cervix	Adenosquamous carcinoma	IB	-	T1bN0M0	D3	0.2207
	Malignant	F	38	Cervix	Adenosquamous carcinoma	IB	-	T1bN0M0	D4	0.2352
	Malignant	F	54	Cervix	Adenosquamous carcinoma	IB	-	T1bN0M0	D5	0.2051
	Malignant	F	54	Cervix	Adenosquamous carcinoma	IB	-	T1bN0M0	D6	0.2278
	Malignant	F	48	Cervix	Adenosquamous carcinoma	III	-	T3N0M0	D7	0.2069
	Malignant	F	48	Cervix	Adenosquamous carcinoma	III	-	T3N0M0	D8	0.2221
	Malignant	F	42	Cervix	Adenosquamous carcinoma	IA	-	T1aN0M0	D9	0.2165
	Malignant	F	42	Cervix	Adenosquamous carcinoma	IA	-	T1aN0M0	D10	0.1972

	Malignant	F	69	Cervix	Squamous cell carcinoma	IB	1	T1bN0M0	E1	0.2072
	Malignant	F	69	Cervix	Squamous cell carcinoma	IB	1	T1bN0M0	E2	0.2262
	Malignant	F	50	Cervix	Squamous cell carcinoma	IIB	1	T2bN0M0	E3	0.3056
	Malignant	F	50	Cervix	Squamous cell carcinoma	IIB	1	T2bN0M0	E4	0.3640
	Malignant	F	53	Cervix	Squamous cell carcinoma	IB2	1--2	T1b2N0M0	E5	0.2122
	Malignant	F	53	Cervix	Squamous cell carcinoma	IB2	1--2	T1b2N0M0	E6	0.2152
	Malignant	F	40	Cervix	Squamous cell carcinoma	I	1--2	T1N0M0	E7	0.2396
	Malignant	F	40	Cervix	Squamous cell carcinoma	I	1--2	T1N0M0	E8	0.2581
	Malignant	F	36	Cervix	Squamous cell carcinoma	IIIB	2	T1bN1M0	E9	0.2083
	Malignant	F	36	Cervix	Squamous cell carcinoma	IIIB	2	T1bN1M0	E10	0.2118
	Malignant	F	33	Cervix	Squamous cell carcinoma	II	2	T2N0M0	F1	0.3140
	Malignant	F	33	Cervix	Squamous cell carcinoma	II	2	T2N0M0	F2	0.2846
	Malignant	F	47	Cervix	Squamous cell carcinoma	II	2	T2N0M0	F3	0.2299
	Malignant	F	47	Cervix	Squamous cell carcinoma	II	2	T2N0M0	F4	0.2514
	Malignant	F	37	Cervix	Squamous cell carcinoma	IB	2	T1bN0M0	F5	0.2148
	Malignant	F	37	Cervix	Squamous cell carcinoma	IB	2	T1bN0M0	F6	0.2458
	Malignant	F	68	Cervix	Squamous cell carcinoma	IB	2	T1bN0M0	F7	0.2300
	Malignant	F	68	Cervix	Squamous cell carcinoma	IB	2	T1bN0M0	F8	0.2423
	Malignant	F	47	Cervix	Squamous cell carcinoma	I	2	T1N0M0	F9	0.2871
	Malignant	F	47	Cervix	Squamous cell carcinoma	I	2	T1N0M0	F10	0.3027
	Malignant	F	62	Cervix	Squamous cell carcinoma	IB	2	T1bN0M0	G1	0.1898
	Malignant	F	62	Cervix	Squamous cell carcinoma	IB	2	T1bN0M0	G2	0.1967
	Malignant	F	39	Cervix	Squamous cell carcinoma	IVA	2	T4N0M0	G3	0.1971
	Malignant	F	39	Cervix	Squamous cell carcinoma	IVA	2	T4N0M0	G4	0.2080
	Malignant	F	27	Cervix	Squamous cell carcinoma	IB	2	T1bN0M0	G5	0.2968
	Malignant	F	27	Cervix	Squamous cell carcinoma	IB	2	T1bN0M0	G6	0.2173
	Malignant	F	32	Cervix	Squamous cell carcinoma	IA	2	T1aN0M0	G7	0.2405
	Malignant	F	32	Cervix	Squamous cell carcinoma	IA	2	T1aN0M0	G8	0.2359
	Malignant	F	76	Cervix	Squamous cell carcinoma	II	2--3	T2N0M0	G9	0.1933
	Malignant	F	76	Cervix	Squamous cell carcinoma	II	2--3	T2N0M0	G10	0.2136
	Malignant	F	43	Cervix	Squamous cell carcinoma	IB1	2--3	T1b1N0M0	H1	0.2370
	Malignant	F	43	Cervix	Squamous cell carcinoma	IB1	2--3	T1b1N0M0	H2	0.2320
	Malignant	F	27	Cervix	Squamous cell carcinoma	IB	3	T1bN0M0	H3	0.2058
	Malignant	F	27	Cervix	Squamous cell carcinoma	IB	3	T1bN0M0	H4	0.1965
	Malignant	F	58	Cervix	Squamous cell carcinoma	IB	3	T1bN0M0	H5	0.2301
	Malignant	F	58	Cervix	Squamous cell carcinoma	IB	3	T1bN0M0	H6	0.2144
	Malignant	F	48	Cervix	Squamous cell carcinoma	IB	3	T1bN0M0	H7	0.2356
	Malignant	F	48	Cervix	Squamous cell carcinoma	IB	3	T1bN0M0	H8	0.2409
	Malignant	F	27	Cervix	Squamous cell carcinoma	IB	3	T1bN0M0	H9	0.2350
	Malignant	F	27	Cervix	Squamous cell carcinoma	IB	3	T1bN0M0	H10	0.2108
	AT	F	57	Cervix	Cancer adjacent cervix tissue	-	-	-	I1	0.2690
	AT	F	57	Cervix	Cancer adjacent cervix tissue	-	-	-	I2	0.2848
	AT	F	50	Cervix	Cancer adjacent cervix tissue	-	-	-	I3	0.3346
	AT	F	50	Cervix	Cancer adjacent cervix tissue	-	-	-	I4	0.1512
	AT	F	71	Cervix	Cancer adjacent cervix tissue	-	-	-	I5	0.2692

	AT	F	71	Cervix	Cancer adjacent cervix tissue	-	-	-	I6	0.2861
	AT	F	62	Cervix	Cancer adjacent cervix tissue	-	-	-	I7	0.3233
	AT	F	62	Cervix	Cancer adjacent cervix tissue	-	-	-	I8	0.1918
	NAT	F	45	Cervix	Adjacent normal cervix tissue	-	-	-	I9	0.2320
	NAT	F	45	Cervix	Adjacent normal cervix tissue	-	-	-	I10	0.2723
	NAT	F	70	Cervix	Adjacent normal cervix tissue	-	-	-	J1	0.2814
	NAT	F	70	Cervix	Adjacent normal cervix tissue	-	-	-	J2	0.2502
	NAT	F	70	Cervix	Adjacent normal cervix tissue	-	-	-	J3	0.2044
	NAT	F	70	Cervix	Adjacent normal cervix tissue	-	-	-	J4	0.2483
	NAT	F	47	Cervix	Adjacent normal cervix tissue	-	-	-	J5	0.2417
	NAT	F	47	Cervix	Adjacent normal cervix tissue	-	-	-	J6	0.1333
	Normal	F	23	Cervix	Cervix tissue	-	-	-	J7	0.2073
	Normal	F	23	Cervix	Cervix tissue	-	-	-	J8	0.1815
	Normal	F	38	Cervix	Cervix tissue	-	-	-	J9	0.4505
	Normal	F	38	Cervix	Cervix tissue	-	-	-	J10	0.3049

Endometrium tissues (catalog no. OD-CT-RpUTR 03-002)

	Tissue type	Sex	Age	Organ	Pathology	Stage	Grade	TNM	Position	PLK5 expression
	tumor	F	62	Uterus	Endometrioid adenocarcinoma	-	G1	T1bM0	A1	0.3211
	tumor	F	62	Uterus	Complex hyperplasia with atypical hyperplasia	-			A2	0.2347
	tumor	F	64	Uterus	Endometrioid adenocarcinoma	-	G1	T1M0	A3	0.1913
	tumor	F	64	Uterus	Endometria	-	G1		A4	0.2097
	tumor	F	53	Uterus	Endometrioid adenocarcinoma	IA	G1	T1aN0M0	A5	0.2519
	tumor	F	53	Uterus	Endometria	-	G1		A6	0.1986
	tumor	F	40	Uterus	Endometrioid adenocarcinoma	3	G2	T3M0	A7	0.2449
	tumor	F	40	Uterus	Endometria	-	G2		A8	0.2818
	tumor	F	66	Uterus	Endometrioid adenocarcinoma	-	G2	T1M0	A9	0.2203
	tumor	F	66	Uterus	Endometria	-	G2		A10	0.2401
	tumor	F	36	Uterus	Endometrioid adenocarcinoma	3	G2	T2N1M0	B1	0.3076
	tumor	F	36	Uterus	Endometria	-	G2		B2	0.3215
	tumor	F	55	Uterus	Endometrioid adenocarcinoma	-	G2	M0	B3	1.4795
	tumor	F	55	Uterus	Endometria	-	G2		B4	0.3295
	tumor	F	88	Uterus	Endometrioid adenocarcinoma	IV	G2	T3M1	B5	0.3307
	tumor	F	88	Uterus	Endometria	-	G2		B6	0.3440
	tumor	F	68	Uterus	Endometrioid adenocarcinoma	I	G2	T1N0M0	B7	0.2465
	tumor	F	68	Uterus	Endometria	-	G2		B8	0.2027
	tumor	F	61	Uterus	Endometrioid adenocarcinoma	I	G2	T1cN0M0	B9	0.2313
	tumor	F	61	Uterus	Endometria	-	G2		B10	0.2376
	tumor	F	53	Uterus	Endometrioid adenocarcinoma	-	G2	M0	C1	0.2867
	tumor	F	53	Uterus	Endometria	-	G2		C2	0.3496
	tumor	F	60	Uterus	Endometrioid adenocarcinoma	I	G2	T1bN0M0	C3	0.2888

	tumor	F	60	Uterus	Endometria	-	G2		C4	0.2615
	tumor	F	79	Uterus	Endometrioid adenocarcinoma	-	G2	M0	C5	0.3769
	tumor	F	79	Uterus	Endometria	-	G2		C6	0.7281
	tumor	F	57	Uterus	Endometrioid adenocarcinoma	-	G2		C7	0.3283
	tumor	F	57	Uterus	Endometria	-	G2		C8	0.2543
	tumor	F	55	Uterus	Mucoid adenocarcinoma	-	G2	N0M0	C9	0.6037
	tumor	F	55	Uterus	Endometria	-	G2		C10	0.3658
	tumor	F	65	Uterus	Endometrioid adenocarcinoma	3	G2	T3M0	D1	0.3371
	tumor	F	65	Uterus	Endometria	-	G2		D2	0.2358
	tumor	F	60	Uterus	Endometrioid adenocarcinoma	I	G2	T1bN0M0	D3	0.2920
	tumor	F	60	Uterus	Endometria	-	G2		D4	0.2264
	tumor	F	62	Uterus	Endometrioid adenocarcinoma	-	G2	M0	D5	0.2719
	tumor	F	62	Uterus	Endometria	-	G2		D6	0.2566
	tumor	F	41	Uterus	Endometrioid adenocarcinoma	-	G2	N0M0	D7	0.4063
	tumor	F	41	Uterus	Kidney tissue	-	G2		D8	0.3561
	tumor	F	56	Uterus	Endometrioid adenocarcinoma	-	G2	N0M0	D9	0.3150
	tumor	F	56	Uterus	Endometria	-	G2		D10	0.2914
	tumor	F	59	Uterus	Endometrioid adenocarcinoma	-	G2	N0M0	E1	0.3289
	tumor	F	59	Uterus	Endometria	-	G2		E2	0.3728
	tumor	F	57	Uterus	Endometrioid adenocarcinoma	-	G2-G3	N0M0	E3	0.2998
	tumor	F	57	Uterus	Endometria	-	G2-G3		E4	0.2968
	tumor	F	53	Uterus	Endometrioid adenocarcinoma	I	G2-G3	T1bN0M0	E5	0.2004
	tumor	F	53	Uterus	Endometria	-	G2-G3		E6	0.2109
	tumor	F	49	Uterus	Endometrioid adenocarcinoma	-	G2-G3	N0M0	E7	0.2700
	tumor	F	49	Uterus	Endometria	-	G2-G3		E8	0.3848
	tumor	F	64	Uterus	Endometrioid adenocarcinoma	I	G3	T1N0M0	E9	0.2156
	tumor	F	64	Uterus	Endometria	-	G3		E10	0.2417
	tumor	F	46	Uterus	Endometrioid adenocarcinoma	3	G3	T3N0M0	F1	0.2725
	tumor	F	46	Uterus	Endometria	-	G3		F2	0.2604
	tumor	F	67	Uterus	Endometrioid adenocarcinoma	-	G3	M0	F3	0.2390
	tumor	F	67	Uterus	Endometria	-	G3		F4	0.5018
	tumor	F	67	Uterus	Endometrioid adenocarcinoma	-	G3	M0	F5	0.2632
	tumor	F	67	Uterus	Endometria	-	G3		F6	0.4349
	tumor	F	67	Uterus	Endometrioid adenocarcinoma	-	G3	M0	F7	0.2274
	tumor	F	67	Uterus	Endometria	-	G3		F8	0.3451
	tumor	F	34	Uterus	Endometrioid adenocarcinoma	-	G3		F9	0.3027
	tumor	F	34	Uterus	Endometria	-	G3		F10	0.0000
	tumor	F	47	Uterus	Endometrioid adenocarcinoma	-	G3	N0M0	G1	0.3405
	tumor	F	47	Uterus	Endometria	-	G3		G2	0.7891
Fallopian tube tissues (catalog no. UTE601)										
	**Tissue type**	**Sex**	**Age**	**Organ**	**Pathology**	**Stage**	**Grade**	**TNM**	**Position**	**PLK5 expression**
	Malignant	F	47	Fallopian tube	Adenocarcinoma	I	2	T1N0M0	A1	0.1627

	Malignant	F	47	Fallopian tube	Adenocarcinoma	I	2	T1N0M0	A2	0.1532
	Malignant	F	46	Fallopian tube	Adenocarcinoma	I	3	T1N0M0	A3	0.1815
	Malignant	F	46	Fallopian tube	Adenocarcinoma	I	3	T1N0M0	A4	0.1561
	Malignant	F	43	Fallopian tube	Adenocarcinoma	II	2--3	T2N0M0	A5	0.2126
	Malignant	F	43	Fallopian tube	Adenocarcinoma	II	2--3	T2N0M0	A6	0.2307
	Malignant	F	52	Fallopian tube	Adenocarcinoma	I	3	T1N0M0	A7	0.1490
	Malignant	F	52	Fallopian tube	Adenocarcinoma	I	3	T1N0M0	A8	0.1508
	Malignant	F	53	Fallopian tube	Adenocarcinoma	IA	3	T1AN0M0	A9	0.1075
	Malignant	F	53	Fallopian tube	Adenocarcinoma	IA	3	T1AN0M0	A10	0.1658
	Malignant	F	60	Fallopian tube	Adenocarcinoma	I	3	T1N0M0	B1	0.0724
	Malignant	F	60	Fallopian tube	Adenocarcinoma	I	3	T1N0M0	B2	0.0694
	Malignant	F	40	Fallopian tube	Adenocarcinoma	I	3	T1N0M0	B3	0.1654
	Malignant	F	40	Fallopian tube	Adenocarcinoma	I	3	T1N0M0	B4	0.1843
	Malignant	F	57	Fallopian tube	Adenocarcinoma	II	3	T2N0M0	B5	0.1233
	Malignant	F	57	Fallopian tube	Adenocarcinoma	II	3	T2N0M0	B6	0.1135
	Malignant	F	51	Fallopian tube	Adenocarcinoma	IA	3	T1AN0M0	B7	0.1594
	Malignant	F	51	Fallopian tube	Adenocarcinoma	IA	3	T1AN0M0	B8	0.1619
	Malignant	F	53	Fallopian tube	Adenocarcinoma	IC	3	T1CN0M0	B9	0.2072
	Malignant	F	53	Fallopian tube	Adenocarcinoma	IC	3	T1CN0M0	B10	0.1949
	Inflammation	F	41	Fallopian tube	Chronic inflammation	-	-	-	C1	0.2028
	Inflammation	F	41	Fallopian tube	Chronic inflammation	-	-	-	C2	0.1463
	Inflammation	F	32	Fallopian tube	Chronic inflammation	-	-	-	C3	0.1577
	Inflammation	F	32	Fallopian tube	Chronic inflammation	-	-	-	C4	0.2682
	Inflammation	F	46	Fallopian tube	Chronic inflammation	-	-	-	C5	0.2551
	Inflammation	F	46	Fallopian tube	Chronic inflammation	-	-	-	C6	0.2709
	Inflammation	F	34	Fallopian tube	Chronic inflammation	-	-	-	C7	0.2682
	Inflammation	F	34	Fallopian tube	Chronic inflammation	-	-	-	C8	0.2825
	Inflammation	F	34	Fallopian tube	Chronic inflammation	-	-	-	C9	0.2447
	Inflammation	F	34	Fallopian tube	Chronic inflammation	-	-	-	C10	0.2152
	Inflammation	F	53	Fallopian tube	Chronic inflammation	-	-	-	D1	0.2279
	Inflammation	F	53	Fallopian tube	Chronic inflammation	-	-	-	D2	0.1962
	Inflammation	F	49	Fallopian tube	Chronic inflammation	-	-	-	D3	0.2368
	Inflammation	F	49	Fallopian tube	Chronic inflammation	-	-	-	D4	0.1926
	Inflammation	F	49	Fallopian tube	Chronic inflammation	-	-	-	D5	0.2262
	Inflammation	F	49	Fallopian tube	Chronic inflammation	-	-	-	D6	0.2274
	Inflammation	F	22	Fallopian tube	Chronic inflammation	-	-	-	D7	0.2260
	Inflammation	F	22	Fallopian tube	Chronic inflammation	-	-	-	D8	0.2315
	Inflammation	F	29	Fallopian tube	Chronic inflammation	-	-	-	D9	0.2545
	Inflammation	F	29	Fallopian tube	Chronic inflammation	-	-	-	D10	0.2959
	Hyperplasia	F	27	Fallopian tube	Chronic inflammation with epithelium hyperplasia	-	-	-	E1	0.1770
	Hyperplasia	F	27	Fallopian tube	Chronic inflammation with epithelium hyperplasia	-	-	-	E2	0.8411
	NAT	F	56	Fallopian tube	Adjacent normal oviductal tissue	-	-	-	E3	0.2001
	NAT	F	56	Fallopian tube	Adjacent normal oviductal tissue	-	-	-	E4	0.1828
	NAT	F	34	Fallopian tube	Adjacent normal oviductal tissue	-	-	-	E5	0.2377

	NAT	F	34	Fallopian tube	Adjacent normal oviductal tissue	-	-	-	E6	0.2602
	NAT	F	26	Fallopian tube	Adjacent normal oviductal tissue	-	-	-	E7	0.2933
	NAT	F	26	Fallopian tube	Adjacent normal oviductal tissue	-	-	-	E8	0.2123
	NAT	F	47	Fallopian tube	Adjacent normal oviductal tissue	-	-	-	E9	0.1722
	NAT	F	47	Fallopian tube	Adjacent normal oviductal tissue	-	-	-	E10	0.1733
	Normal	F	21	Fallopian tube	Oviductal tissue	-	-	-	F1	0.1599
	Normal	F	21	Fallopian tube	Oviductal tissue	-	-	-	F2	0.1544
	Normal	F	15	Fallopian tube	Oviductal tissue	-	-	-	F3	0.2172
	Normal	F	15	Fallopian tube	Oviductal tissue	-	-	-	F4	0.2097
	Normal	F	18	Fallopian tube	Oviductal tissue	-	-	-	F5	0.1931
	Normal	F	18	Fallopian tube	Oviductal tissue	-	-	-	F6	0.2003
	Normal	F	21	Fallopian tube	Oviductal tissue	-	-	-	F7	0.2343
	Normal	F	21	Fallopian tube	Oviductal tissue	-	-	-	F8	0.2219
	Normal	F	21	Fallopian tube	Oviductal tissue	-	-	-	F9	0.2245
	Normal	F	21	Fallopian tube	Oviductal tissue	-	-	-	F10	0.2404

Lung tissues (catalog no. BC04002a)

	**Tissue type**	**Sex**	**Age**	**Organ**	**Pathology**	**Stage**	**Grade**	**TNM**	**Position**	**PLK5 expression**
	Malignant	M	65	Lung	Squamous cell carcinoma	IIIA	*	T3N1M0	A1	0.2776
	Malignant	M	46	Lung	Squamous cell carcinoma with necrosis (sparse)	IIIA	-	T3N1M0	A2	0.3898
	Malignant	M	65	Lung	Squamous cell carcinoma with necrosis	IIB	2	T3N0M0	A3	0.3921
	Malignant	M	68	Lung	Squamous cell carcinoma	IIA	2	T2N1M0	A4	0.2570
	Malignant	M	50	Lung	Squamous cell carcinoma	IIIA	2	T3N1M0	A5	0.2590
	Malignant	M	57	Lung	Squamous cell carcinoma	IIIA	2	T3N2M0	A6	0.2402
	Malignant	M	51	Lung	Squamous cell carcinoma	IB	2	T2N0M0	A7	0.2006
	Malignant	M	57	Lung	Squamous cell carcinoma	IB	2	T2N0M0	A8	0.1523
	Malignant	F	68	Lung	Squamous cell carcinoma	IIA	2	T2N1M0	A9	0.2101
	Malignant	M	69	Lung	Squamous cell carcinoma	IB	2	T2N0M0	A10	0.1869
	Malignant	M	69	Lung	Squamous cell carcinoma	IIIA	2	T2N2M0	B1	0.3044
	Malignant	F	43	Lung	Squamous cell carcinoma	IIA	2	T2N1M0	B2	0.3324
	Malignant	M	47	Lung	Squamous cell carcinoma	IA	1	T1N0M0	B3	0.2484
	Malignant	M	55	Lung	Squamous cell carcinoma	IB	2	T2N0M0	B4	0.2849
	Malignant	M	41	Lung	Squamous cell carcinoma	IIIA	2	T2N2M0	B5	0.2662
	Malignant	M	53	Lung	Squamous cell carcinoma	IIA	2	T2N1M0	B6	0.2802
	Malignant	F	50	Lung	Squamous cell carcinoma	IB	2	T2N0M0	B7	0.2544
	Malignant	M	66	Lung	Squamous cell carcinoma	IB	2	T2N0M0	B8	0.2802
	Malignant	M	44	Lung	Squamous cell carcinoma	IIA	2	T2N1M0	B9	0.2350
	Malignant	M	60	Lung	Squamous cell carcinoma	IB	3	T2N0M0	B10	0.2328
	Malignant	M	70	Lung	Adenocarcinoma	IA	1	T1N0M0	C1	0.2477
	Malignant	F	62	Lung	Adenocarcinoma	IIA	1	T2N1M0	C2	0.3499
	Malignant	F	53	Lung	Adenocarcinoma	IA	2	T1N0M0	C3	0.3279
	Malignant	F	43	Lung	Adenocarcinoma	IIIA	2	T2N2M0	C4	0.3401
	Malignant	F	44	Lung	Adenocarcinoma	IIIA	2	T3N1M0	C5	0.3498

	Malignant	M	50	Lung	Adenocarcinoma	IB	2	T2N0M0	C6	0.3759
	Malignant	M	63	Lung	Adenocarcinoma	IIIA	2	T3N2M0	C7	0.2823
	Malignant	F	46	Lung	Adenocarcinoma	IA	2	T1N0M0	C8	0.2991
	Malignant	F	61	Lung	Adenocarcinoma	IIIA	2	T3N1M0	C9	0.2901
	Malignant	M	54	Lung	Adenocarcinoma	IIIA	2	T4N0M0	C10	0.5798
	Malignant	F	63	Lung	Adenocarcinoma	IB	2	T2N0M0	D1	0.2681
	Malignant	M	50	Lung	Adenocarcinoma	IIIA	2	T3N1M0	D2	0.2822
	Malignant	F	44	Lung	Adenocarcinoma	IIA	2	T2N1M0	D3	0.3041
	Malignant	M	56	Lung	Adenocarcinoma	IIB	2	T3N0M0	D4	0.2864
	Malignant	M	42	Lung	Adenocarcinoma	IIIA	2	T3N1M0	D5	0.2557
	Malignant	M	50	Lung	Adenocarcinoma	IIA	3	T2N1M0	D6	0.2792
	Malignant	M	67	Lung	Adenocarcinoma	IB	3	T2N0M0	D7	0.3097
	Malignant	M	53	Lung	Adenocarcinoma	IB	2	T2N0M0	D8	0.2653
	Malignant	M	57	Lung	Adenocarcinoma	IB	3	T2N0M0	D9	0.3008
	Malignant	M	59	Lung	Adenocarcinoma	IIA	2	T2N1M0	D10	0.3063
	Malignant	F	51	Lung	Small cell undifferentiated carcinoma	IA	-	T1N0M0	E1	0.1624
	Malignant	M	39	Lung	Small cell undifferentiated carcinoma	IIA	-	T2N1M0	E2	0.2021
	Malignant	F	42	Lung	Small cell undifferentiated carcinoma	IIIA	-	T4N1M0	E3	0.2028
	Malignant	M	73	Lung	Small cell undifferentiated carcinoma	IIIA	-	T3N1M0	E4	0.2662
	Malignant	F	66	Lung	Small cell undifferentiated carcinoma	IA	-	T1N0M0	E5	0.3732
	Malignant	F	65	Lung	Small cell undifferentiated carcinoma	IA	-	T1N0M0	E6	0.2535
	Malignant	M	54	Lung	Small cell undifferentiated carcinoma	IIA	-	T2N1M0	E7	0.1411
	Malignant	M	52	Lung	Small cell undifferentiated carcinoma	IIIA	-	T2N2M0	E8	0.2305
	Malignant	M	60	Lung	Small cell undifferentiated carcinoma	IIIA	-	T3N1M0	E9	0.1785
	Malignant	M	60	Lung	Small cell undifferentiated carcinoma	IB	-	T2N0M0	E10	0.1663
	Malignant	M	53	Lung	Invasive adenocarcinoma	IIA	-	T2N1M0	F1	0.1531
	Malignant	M	41	Lung	Invasive adenocarcinoma	IA	-	T1N0M0	F2	0.2768
	Malignant	M	50	Lung	Invasive adenocarcinoma	IB	-	T2N0M0	F3	0.4776
	Malignant	M	56	Lung	Invasive adenocarcinoma	IB	-	T2N0M0	F4	0.3369
	Malignant	F	48	Lung	Invasive adenocarcinoma	IA	-	T1N0M0	F5	0.3374
	Malignant	F	66	Lung	Invasive adenocarcinoma	IA	-	T1N0M0	F6	0.3116
	Malignant	F	67	Lung	Invasive adenocarcinoma	IA	-	T1N0M0	F7	0.2322
	Malignant	F	60	Lung	Invasive adenocarcinoma	IA	-	T1N0M0	F8	0.2770
	Malignant	M	60	Lung	Invasive adenocarcinoma with necrosis	IIA	-	T2N1M0	F9	0.3405
	Malignant	F	50	Lung	Invasive adenocarcinoma	IB	-	T2N0M0	F10	0.3017
	Malignant	M	60	Lung	Atypical carcinoid	IA	-	T1N0M0	G1	0.1431
	Malignant	F	36	Lung	Atypical carcinoid	IB	-	T2N0M0	G2	0.1424
	Malignant	M	55	Lung	Atypical carcinoid	IB	*	T2N0M0	G3	0.4135
	Malignant	M	55	Lung	Atypical carcinoid	IIA	-	T2N1M0	G4	0.3028
	Malignant	M	59	Lung	Atypical carcinoid	IB	-	T2N0M0	G5	0.1847

	Metastasis	M	60	Lymph node	Metastatic squamous cell carcinoma from lung	-	-	-	G6	0.4720
	Metastasis	M	62	Lymph node	Metastatic squamous cell carcinoma from lung	-	-	-	G7	0.2622
	Metastasis	M	64	Lymph node	Metastatic squamous cell carcinoma from lung	-	-	-	G8	0.2823
	Metastasis	M	54	Lymph node	Metastatic squamous cell carcinoma from lung	-	-	-	G9	0.3294
	Metastasis	M	45	Lymph node	Metastatic squamous cell carcinoma from lung	-	-	-	G10	0.2392
	Metastasis	M	32	Lymph node	Metastatic squamous cell carcinoma from lung	-	-	-	H1	0.2849
	Metastasis	M	58	Lymph node	Metastatic squamous cell carcinoma from lung	-	-	-	H2	0.2570
	Metastasis	M	60	Lymph node	Metastatic squamous cell carcinoma from lung	-	-	-	H3	0.3176
	Metastasis	M	59	Lymph node	Metastatic squamous cell carcinoma from lung (fibrous tissue and blood vessel)	-	*	-	H4	0.2397
	Metastasis	M	52	Lymph node	Metastatic squamous cell carcinoma from lung	-	-	-	H5	0.3429
	Benign	M	59	Lung	Inflammatory pseudotumor	-	-	-	H6	0.2946
	Benign	M	67	Lung	Inflammatory pseudotumor	-	-	-	H7	0.3072
	Benign	M	30	Lung	Inflammatory pseudotumor	-	-	-	H8	0.3513
	Benign	F	39	Lung	Inflammatory pseudotumor	-	-	-	H9	0.2163
	Benign	F	37	Lung	Inflammatory pseudotumor	-	-	-	H10	0.3431
	Benign	F	48	Lung	Tuberculosis	-	-	-	I1	0.3922
	Benign	M	35	Lung	Tuberculosis	-	-	-	I2	0.3310
	Benign	M	48	Lung	Tuberculosis	-	-	-	I3	0.3231
	Benign	M	63	Lung	Tuberculosis	-	-	-	I4	0.3375
	Benign	M	67	Lung	Tuberculosis	-	*	-	I5	0.4920
	AT	M	49	Lung	Cancer adjacent lung tissue	-	-	-	I6	0.4447
	AT	F	53	Lung	Cancer adjacent lung tissue	-	-	-	I7	0.3948
	AT	M	39	Lung	Cancer adjacent lung tissue	-	-	-	I8	0.2293
	AT	M	42	Lung	Cancer adjacent lung tissue	-	-	-	I9	0.4237
	AT	M	66	Lung	Cancer adjacent lung tissue	-	-	-	I10	0.5051
	NAT	M	54	Lung	Adjacent normal lung tissue	-	-	-	J1	0.5104
	NAT	M	64	Lung	Adjacent normal lung tissue	-	-	-	J2	0.4041
	NAT	F	36	Lung	Adjacent normal lung tissue of No.62	-	-	-	J3	0.4114
	NAT	M	47	Lung	Adjacent normal lung tissue	-	-	-	J4	0.4904
	NAT	F	51	Lung	Adjacent normal lung tissue	-	-	-	J5	0.4677
	Normal	M	24	Lung	Lung tissue	-	-	-	J6	0.1674
	Normal	F	14	Lung	Lung tissue	-	-	-	J7	0.5058
	Normal	F	21	Lung	Lung tissue	-	-	-	J8	0.3737
	Normal	M	42	Lung	Lung tissue	-	-	-	J9	0.3855
	Normal	F	15	Lung	Lung tissue	-	-	-	J10	0.4500
Normal ovarian tissues (catalog no. OV806)										
	**Tissue type**	**Sex**	**Age**	**Organ**	**Pathology**	**Stage**	**Grade**	**TNM**	**Position**	**PLK5 expression**
	normal	F	50	Ovary	Normal ovarian tissue	-			A1	0.0000

	normal	F	50	Ovary	Normal ovarian tissue	-			A2	0.2927
	normal	F	63	Ovary	Normal ovarian tissue	-			A3	0.3819
	normal	F	63	Ovary	Normal ovarian tissue	-			A4	0.5813
	normal	F	39	Ovary	Normal ovarian tissue	-			A5	0.3669
	normal	F	39	Ovary	Normal ovarian tissue	-			A6	0.3106
	normal	F	29	Ovary	Normal ovarian tissue	-			A7	0.3120
	normal	F	29	Ovary	Normal ovarian tissue	-			A8	0.1916
	normal	F	40	Ovary	Normal ovarian tissue	-			A9	0.2578
	normal	F	40	Ovary	Normal ovarian tissue	-			A10	0.2515
	normal	F	48	Ovary	Normal ovarian tissue	-			B1	0.2237
	normal	F	48	Ovary	Normal ovarian tissue	-			B2	0.3447
	normal	F	63	Ovary	Normal ovarian tissue	-			B3	0.2534
	normal	F	63	Ovary	Normal ovarian tissue	-			B4	0.2577
	normal	F	36	Ovary	Normal ovarian tissue	-			B5	0.2548
	normal	F	36	Ovary	Normal ovarian tissue	-			B6	0.2512
	normal	F	45	Ovary	Normal ovarian tissue	-			B7	0.1712
	normal	F	45	Ovary	Normal ovarian tissue	-			B8	0.3062
	normal	F	45	Ovary	Normal ovarian tissue	-			B9	0.2587
	normal	F	45	Ovary	Normal ovarian tissue	-			B10	0.3151
	normal	F	53	Ovary	Normal ovarian tissue	-			C1	0.0000
	normal	F	53	Ovary	Normal ovarian tissue	-			C2	0.2365
	normal	F	42	Ovary	Normal ovarian tissue	-			C3	0.2551
	normal	F	42	Ovary	Normal ovarian tissue	-			C4	0.3632
	normal	F	40	Ovary	Normal ovarian tissue	-			C5	0.2895
	normal	F	40	Ovary	Normal ovarian tissue	-			C6	0.3578
	normal	F	48	Ovary	Normal ovarian tissue	-			C7	0.3866
	normal	F	48	Ovary	Normal ovarian tissue	-			C8	0.3708
	normal	F	38	Ovary	Normal ovarian tissue	-			C9	0.4201
	normal	F	38	Ovary	Normal ovarian tissue (with germinal epithelium invagination)	-			C10	0.3984
	normal	F	22	Ovary	Normal ovarian tissue	-			D1	0.6976
	normal	F	22	Ovary	Normal ovarian tissue	-			D2	0.4409
	normal	F	40	Ovary	Normal ovarian tissue	-			D3	0.3079
	normal	F	40	Ovary	Normal ovarian tissue	-			D4	0.3005
	normal	F	33	Ovary	Normal ovarian tissue	-			D5	0.2624
	normal	F	33	Ovary	Normal ovarian tissue	-			D6	0.2680
	normal	F	57	Ovary	Normal ovarian tissue	-			D7	0.2037
	normal	F	57	Ovary	Normal ovarian tissue	-			D8	0.2034
	normal	F	41	Ovary	Normal ovarian tissue	-			D9	0.2866
	normal	F	41	Ovary	Normal ovarian tissue	-			D10	0.3084
	normal	F	69	Ovary	Normal ovarian tissue	-			E1	0.2615
	normal	F	69	Ovary	Normal ovarian tissue	-			E2	0.2623
	normal	F	38	Ovary	Normal ovarian tissue	-			E3	0.3701
	normal	F	38	Ovary	Normal ovarian tissue	-			E4	0.3543
	normal	F	49	Ovary	Normal ovarian tissue	-			E5	0.3828
	normal	F	49	Ovary	Normal ovarian tissue	-			E6	0.4021

	normal	F	49	Ovary	Normal ovarian tissue	-			E7	0.3609
	normal	F	49	Ovary	Normal ovarian tissue	-			E8	0.4538
	normal	F	50	Ovary	Normal ovarian tissue	-			E9	0.2763
	normal	F	50	Ovary	Normal ovarian tissue	-			E10	0.5019
	normal	F	48	Ovary	Normal ovarian tissue	-			F1	0.1928
	normal	F	48	Ovary	Normal ovarian tissue	-			F2	0.2521
	normal	F	42	Ovary	Normal ovarian tissue	-			F3	0.3407
	normal	F	42	Ovary	Normal ovarian tissue	-			F4	0.3971
	normal	F	62	Ovary	Normal ovarian tissue	-			F5	0.1770
	normal	F	62	Ovary	Normal ovarian tissue	-			F6	0.2077
	normal	F	41	Ovary	Normal ovarian tissue	-			F7	0.2137
	normal	F	41	Ovary	Normal ovarian tissue	-			F8	0.2094
	normal	F	53	Ovary	Normal ovarian tissue	-			F9	0.3463
	normal	F	53	Ovary	Normal ovarian tissue	-			F10	0.3969

Disease spectrum ovarian tissues (catalog no. OV1005b)

	**Tissue type**	**Sex**	**Age**	**Organ**	**Pathology**	**Stage**	**Grade**	**TNM**	**Position**	**PLK5 expression**
	Malignant	F	65	Ovary	Low grade serous carcinoma	I	-	T1N0M0	A1	0.1049
	Malignant	F	38	Ovary	Low grade serous carcinoma	IIIC	-	T3cN1M0	A2	0.1644
	Malignant	F	51	Ovary	High grade serous carcinoma	IIIC	-	T3cN1M0	A3	0.1841
	Malignant	F	22	Ovary	High grade serous carcinoma	IIB	-	T2bN0M0	A4	0.0020
	Malignant	F	48	Ovary	Low grade serous carcinoma	I	-	T1N0M0	A5	0.0010
	Malignant	F	26	Ovary	High grade serous carcinoma	IIIC	-	T3cN1M0	A6	0.0011
	Malignant	F	25	Ovary	Low grade serous carcinoma	I	-	T1N0M0	A7	0.0022
	Malignant	F	50	Ovary	High grade serous carcinoma	II	*	T2N0M0	A8	0.0032
	Malignant	F	26	Ovary	High grade serous carcinoma	IC	-	T1cN0M0	A9	0.0031
	Malignant	F	47	Ovary	High grade serous carcinoma	I	-	T1N0M0	A10	0.0004
	Malignant	F	58	Ovary	High grade serous carcinoma	I	-	T1N0M0	B1	0.2622
	Malignant	F	57	Ovary	High grade serous carcinoma	IC	-	T1cN0M0	B2	0.2525
	Malignant	F	51	Ovary	High grade serous carcinoma	IA	*	T1aN0M0	B3	0.2364
	Malignant	F	52	Ovary	High grade serous carcinoma	II	-	T2N0M0	B4	0.2400
	Malignant	F	54	Ovary	High grade serous carcinoma	IIIC	-	T3cN1M0	B5	0.1947
	Malignant	F	33	Ovary	High grade serous carcinoma	I	-	T1N0M0	B6	0.1562
	Malignant	F	56	Ovary	High grade serous carcinoma	II	-	T2N0M0	B7	0.0556
	Malignant	F	41	Ovary	High grade serous carcinoma	I	-	T1N0M0	B8	0.0318
	Malignant	F	46	Ovary	High grade serous carcinoma	III	-	T3aN0M0	B9	0.1240
	Malignant	F	46	Ovary	High grade serous carcinoma	IIIC	*	T2cN1M0	B10	0.1626
	Malignant	F	57	Ovary	High grade serous carcinoma	IIIC	-	T3cN1M0	C1	0.2620
	Malignant	F	75	Ovary	High grade serous carcinoma	II	-	T2N0M0	C2	0.2674
	Malignant	F	54	Ovary	High grade serous carcinoma	IIIC	-	T3cN1M0	C3	0.2628
	Malignant	F	49	Ovary	High grade serous carcinoma	II	-	T2N0M0	C4	0.2436

	Malignant	F	50	Ovary	High grade serous carcinoma	I	-	T1N0M0	C5	0.3092
	Malignant	F	52	Ovary	High grade serous carcinoma	II	*	T2N0M0	C6	0.3751
	Malignant	F	47	Ovary	High grade serous carcinoma	IIIC	-	T3cN1M0	C7	0.1907
	Malignant	F	34	Ovary	Mucinous adenocarcinoma	IB	2	T1bN0M0	C8	0.2455
	Malignant	F	63	Ovary	Mucinous adenocarcinoma	IA	1	T1aN0M0	C9	0.2941
	Malignant	F	69	Ovary	Mucinous adenocarcinoma	IB	1	T1bN0M0	C10	0.2908
	Malignant	F	46	Ovary	Endometrioid adenocarcinoma	II	1--2	T2N0M0	D1	0.2762
	Malignant	F	47	Ovary	Endometrioid adenocarcinoma	IIA	1--2	T2aN0M0	D2	0.2597
	Malignant	F	54	Ovary	Endometrioid adenocarcinoma	IB	1--2	T1bN0M0	D3	0.2614
	Malignant	F	65	Ovary	Adenocarcinoma (sparse)	IC	2	T1cN0M0	D4	0.3242
	Malignant	F	55	Ovary	Endometrioid adenocarcinoma	I	2	T1N0M0	D5	0.2938
	Malignant	F	54	Ovary	Endometrioid adenocarcinoma	IB	1	T1bN0M0	D6	0.2622
	Malignant	F	43	Ovary	Endometrioid adenocarcinoma	IC	2	T1cN0M0	D7	0.2405
	Malignant	F	55	Ovary	Endometrioid adenocarcinoma with necrosis	I	2	T1N0M0	D8	0.3168
	Malignant	F	53	Ovary	Endometrioid adenocarcinoma	IIA	3	T2aN0M0	D9	0.2357
	Malignant	F	50	Ovary	Endometrioid adenocarcinoma	IIIC	2	T3bN1M0	D10	0.2639
	Malignant	F	51	Ovary	Invasive urothelial carcinoma	IB	-	T1bN0M0	E1	0.2869
	Malignant	F	39	Ovary	Invasive urothelial carcinoma	IA	-	T1aN0M0	E2	0.2432
	Malignant	F	38	Ovary	Invasive urothelial carcinoma	I	-	T1N0M0	E3	0.2890
	Malignant	F	66	Ovary	Invasive urothelial carcinoma with squamous metaplasia	IA	-	T1aN0M0	E4	0.1994
	Malignant	F	53	Ovary	Invasive urothelial carcinoma	I	-	T1N0M0	E5	0.2212
	Metastasis	F	47	Mesentery	Metastatic papillary adenocarcinoma from ovary	-	2	-	E6	0.2643
	Metastasis	F	57	Greater omentum	Metastatic papillary adenocarcinoma from ovary	-	2	-	E7	0.2524
	Metastasis	F	65	Greater omentum	Metastatic papillary adenocarcinoma from ovary	-	2	-	E8	0.2621
	Metastasis	F	59	Mesentery	Metastatic papillary adenocarcinoma from ovary	-	2	-	E9	0.3232
	Metastasis	F	28	Greater omentum	Metastatic papillary adenocarcinoma with calcification from ovary	-	2	-	E10	0.3044
	Metastasis	F	64	Greater omentum	Metastatic papillary adenocarcinoma from ovary	-	2	-	F1	0.2520
	Metastasis	F	50	Greater omentum	Metastatic papillary adenocarcinoma from ovary	-	2	-	F2	0.2610
	Metastasis	F	58	Greater omentum	Metastatic adenocarcinoma from ovary	-	3	-	F3	0.2373
	Metastasis	F	47	Peritoneum	Metastatic adenocarcinoma from ovary	-	3	-	F4	0.2808
	Metastasis	F	49	Peritoneum	Metastatic adenocarcinoma from ovary	-	3	-	F5	0.2539
	Borderline	F	34	Ovary	Borderline serous papillary cystadenoma	-	-	-	F6	0.2810

	Borderline	F	34	Ovary	Borderline serous papillary cystadenoma	-	-	-	F7	0.2954
	Borderline	F	28	Ovary	Borderline serous papillary cystadenoma	-	-	-	F8	0.3052
	Borderline	F	22	Ovary	Borderline mucinous papillary cystadenoma	-	-	-	F9	0.3387
	Borderline	F	60	Ovary	Borderline serous papillary cystadenoma	-	-	-	F10	0.2786
	Borderline	F	50	Ovary	Borderline serous papillary cystadenoma	-	-	-	G1	0.4095
	Borderline	F	37	Ovary	Borderline mucinous papillary cystadenoma	-	-	-	G2	0.2688
	Benign	F	62	Ovary	Serous cystadenoma	-	-	-	G3	0.3315
	Benign	F	70	Ovary	Serous cystadenoma	-	-	-	G4	0.3322
	Benign	F	49	Ovary	Serous cystadenoma	-	-	-	G5	0.3706
	Benign	F	16	Ovary	Serous cystadenoma	-	*	-	G6	0.4224
	Benign	F	34	Ovary	Serous cystadenoma	-	-	-	G7	0.3632
	Benign	F	22	Ovary	Serous cystadenoma	-	-	-	G8	0.3305
	Benign	F	19	Ovary	Mucinous cystadenoma	-	-	-	G9	0.2775
	Benign	F	17	Ovary	Mucinous cystadenoma	-	-	-	G10	0.3292
	Benign	F	41	Uterus	Mucinous cystadenoma	-	-	-	H1	0.3893
	Benign	F	26	Ovary	Mucinous cystadenoma	-	-	-	H2	0.3588
	Benign	F	22	Ovary	Mucinous cystadenoma	-	*	-	H3	0.3096
	Benign	F	38	Ovary	Mucinous cystadenoma	-	*	-	H4	0.3410
	Benign	F	47	Ovary	Mucinous cystadenoma	-	-	-	H5	0.2717
	Benign	F	70	Ovary	Mucinous cystadenoma	-	-	-	H6	0.3633
	Benign	F	51	Ovary	Mucinous cystadenoma	-	-	-	H7	0.2699
	Benign	F	29	Ovary	Mucinous cystadenoma	-	-	-	H8	0.3114
	Benign	F	35	Ovary	Mucinous cystadenoma	-	-	-	H9	0.4033
	Benign	F	18	Ovary	Mucinous cystadenoma	-	-	-	H10	0.2560
	NAT	F	30	Ovary	Adjacent normal ovary tissue	-	-	-	I1	0.3343
	NAT	F	39	Ovary	Adjacent normal ovary tissue	-	-	-	I2	0.4313
	NAT	F	29	Ovary	Adjacent normal ovary tissue	-	-	-	I3	0.0000
	NAT	F	41	Ovary	Adjacent normal ovary tissue	-	-	-	I4	0.3388
	NAT	F	62	Ovary	Adjacent normal ovary tissue	-	-	-	I5	0.0000
	NAT	F	63	Ovary	Adjacent normal ovary tissue	-	-	-	I6	0.4095
	NAT	F	45	Ovary	Adjacent normal ovary tissue	-	-	-	I7	0.2841
	NAT	F	48	Ovary	Adjacent normal ovary tissue	-	-	-	I8	0.4510
	NAT	F	53	Ovary	Adjacent normal ovary tissue	-	-	-	I9	0.4332
	NAT	F	53	Ovary	Adjacent normal ovary tissue	-	-	-	I10	0.1679
	NAT	F	57	Ovary	Adjacent normal ovary tissue	-	-	-	J1	0.0000
	NAT	F	38	Ovary	Adjacent normal ovary tissue	-	-	-	J2	0.3718
	NAT	F	53	Ovary	Adjacent normal ovary tissue	-	-	-	J3	0.3668
	NAT	F	59	Ovary	Adjacent normal ovary tissue	-	-	-	J4	0.2453
	NAT	F	48	Ovary	Adjacent normal ovary tissue	-	-	-	J5	0.2692
	NAT	F	50	Ovary	Adjacent normal ovary tissue	-	-	-	J6	0.3174

	NAT	F	52	Ovary	Adjacent normal ovary tissue	-	-	-	J7	0.1852
	Normal	F	27	Ovary	Ovary tissue	-	*	-	J8	0.0000
	Normal	F	34	Ovary	Ovary tissue	-	*	-	J9	0.0000
	Normal	F	19	Ovary	Ovary tissue	-	-	-	J10	0.3091
Primary ovarian cancer tissues (UW BioBank TMA)										
	**Tissue type**	**Sex**	**Age**	**Organ**	**Pathology**	**Stage**	**Grade**	**TNM**	**Position**	**PLK5 expression**
	Malignant	F	76	Ovary	PS	IIIC omental	3	-	1; 1	0.2533
	Malignant	F	59	Ovary	Mucinous	IIIC omental and LN	3	-	2; 1	0.2449
	Malignant	F	65	Ovary	PS	IC	2	-	3; 1	0.2577
	Malignant	F	48	Ovary	PS	IIIC omental and LN	3	-	4; 1	0.2686
	Malignant	F	51	Ovary	PS	IIC	3	-	5; 1	0.2672
	Malignant	F	72	Ovary	PS	IIIC omental	3	-	6; 1	0.2310
	Malignant	F	82	Ovary	PS	IIIC omental	3	-	7; 1	0.3155
	Malignant	F	38	Ovary	PS	IIIC LN	1	-	8; 1	0.3958
	Malignant	F	58	Ovary	PS	IC	1	-	9; 1	0.2495
	Malignant	F	61	Ovary	Clear	IIIC LN	3	-	10; 1	0.2425
	Malignant	F	81	Ovary	PS	IA	3	-	11; 1	0.4076
	Malignant	F	79	Ovary	PS	IIIC omental	3	-	12; 1	0.2464
	Malignant	F	44	Ovary	Mucinous	IIIB	3	-	13; 1	0.2667
	Malignant	F	79	Ovary	PS	IIIB	3	-	14; 1	0.2597
	Malignant	F	57	Ovary	Endometroid	IIIC omental	3	-	15; 1	0.2227
	Malignant	F	44	Ovary	PS	IIIC omental	3	-	16; 1	0.2539
	Metastasis	F	67	Ovary	PS	IV	3	-	17; 1	0.2404
	Metastasis	F	77	Ovary	PS	IV	3	-	18; 1	0.2679
	Malignant	F	60	Ovary	PS	IIIC omental	3	-	19; 1	0.2170
	Malignant	F	65	Ovary	Other	IIIA	3	-	20; 1	0.1775
	Malignant	F	64	Ovary	PS	IIIC omental	3	-	21; 1	0.2277
	Malignant	F	61	Ovary	Endometroid	IIIC omental	2	-	22; 1	0.5167
	Malignant	F	44	Ovary	PS	IA	1	-	22; 2	0.2493
	Malignant	F	50	Ovary	PS	IIIC omental	3	-	21; 2	0.2825
	Malignant	F	74	Ovary	Endometroid	IIIC omental	3	-	20; 2	0.2453
	Malignant	F	40	Ovary	PS	IC	1	-	19; 2	0.2455
	Malignant	F	53	Ovary	PS	IIIC omental and LN	3	-	18; 2	0.2352
	Metastasis	F	44	Ovary	Endometroid	IV	3	-	17; 2	0.2662
	Malignant	F	38	Ovary	PS	IIB	1	-	16; 2	0.4960
	Malignant	F	62	Fallopian tube	PS	IIA	2	-	15; 2	0.2535

	Malignant	F	77	Ovary	PS	IIIC omental	3	-	14; 2	0.2444
	Malignant	F	48	Ovary	PS	IIIC omental	3	-	13; 2	0.0000
	Malignant	F	52	Ovary	PS	IIIC omental	2	-	12; 2	0.2781
	Malignant	F	44	Ovary	PS	IIIC omental	3	-	11; 2	0.4542
	Malignant	F	47	Ovary	PS	IIIC omental	3	-	10; 2	0.2447
	Malignant	F	74	Ovary	PS	IIIC omental	3	-	9; 2	0.2137
	Malignant	F	58	Ovary	PS	IIIC omental and LN	3	-	8; 2	0.2256
	Malignant	F	69	Ovary	Clear	IIIC omental	3	-	7; 2	0.2357
	Malignant	F	55	Ovary	PS	IIC	3	-	6; 2	0.1943
	Malignant	F	63	Ovary	PS	IIIC omental	3	-	5; 2	0.2285
	Metastasis	F	49	Ovary	PS	IV	2	-	4; 2	0.2419
	Malignant	F	84	Ovary	PS	IIIC omental	3	-	3; 2	0.2616
	Malignant	F	73	Ovary	Endometroid	IIIC omental	2	-	2; 2	0.2222
	Malignant	F	46	Ovary	Clear	IC	1	-	1; 2	0.2073
	Metastasis	F	66	Ovary	Clear	IV	3	-	1; 3	0.2477
	Malignant	F	70	Ovary	PS	IIIC omental	3	-	2; 3	0.2950
	Malignant	F	68	Ovary	PS	IIA	3	-	3; 3	0.1816
	Malignant	F	48	Fallopian tube	PS	IIIB	3	-	4; 3	0.2536
	Malignant	F	68	Ovary	PS	IIIC omental	3	-	5; 3	0.3104
	Malignant	F	78	Ovary	PS	IIIC omental	3	-	6; 3	0.2309
	Malignant	F	64	Ovary	PS	IIIC omental	3	-	7; 3	0.2489
	Malignant	F	42	Ovary	PS	IIIC omental	3	-	8; 3	0.2274
	Malignant	F	75	Ovary	Endometroid	IIIC omental	3	-	9; 3	0.3127
	Malignant	F	54	Ovary	Adeno NOS	IC	2	-	10; 3	0.2673
	Malignant	F	73	Ovary	Adeno NOS	IIIC omental	3	-	11; 3	0.2360
	Malignant	F	68	Ovary	Endometroid	IIC	3	-	12; 3	0.2185
	Malignant	F	55	Ovary	PS	IC	2	-	13; 3	0.2061
	Malignant	F	44	Ovary	Other	IIIC LN	3	-	14; 3	0.2687
	Malignant	F	50	Ovary	PS	IIIC LN	3	-	15; 3	0.2525
	Malignant	F	63	Ovary	PS	IIIC omental and LN	3	-	16; 3	0.2392
	Malignant	F	50	Ovary	PS	IIIC omental	3	-	17; 3	0.2410
	Malignant	F	65	Ovary	Endometroid	IIIC omental	3	-	18; 3	0.2436
	Malignant	F	57	Ovary	Endometroid	IIC	3	-	19; 3	0.2163
	Malignant	F	83	Ovary	PS	IA	2	-	20; 3	0.2008

	Malignant	F	54	Ovary	PS	IIIC omental	3	-	21; 3	0.2090
	Malignant	F	66	Ovary	Endometroid	IIIC omental	3	-	22; 3	0.2199
	Malignant	F	70	Ovary	Endometroid	IA	1	-	22; 4	0.0000
	Malignant	F	49	Ovary	PS	IA	1	-	21; 4	0.2587
	Malignant	F	67	Ovary	PS	IIIC omental	3	-	20; 4	0.2373
	Malignant	F	52	Ovary	PS	IIA	3	-	19; 4	0.2307
	Metastasis	F	56	Ovary	PS	IV	3	-	18; 4	0.2483
	Malignant	F	58	Ovary	PS	IIIC omental	3	-	17; 4	0.1959
	Malignant	F	66	Ovary	PS	IIIC omental	3	-	16; 4	0.2219
	Malignant	F	74	Ovary	PS	IIIC omental	3	-	15; 4	0.3155
	Malignant	F	58	Ovary	Mucinous	IIIC omental	2	-	14; 4	0.0000
	Metastasis	F	15	Ovary	PS	IV	2	-	13; 4	0.3328
	Malignant	F	44	Ovary	Mucinous	IIIC omental and LN	3	-	12; 4	0.2811
	Malignant	F	85	Ovary	PS	IA	1	-	11; 4	0.3036
	Malignant	F	46	Ovary	PS	IIIB	2	-	10; 4	0.3003
	Malignant	F	44	Ovary	PS	IIIC omental	3	-	9; 4	0.2405
	Malignant	F	47	Ovary	PS	IIIA	3	-	8; 4	0.2339
	Malignant	F	73	Ovary	PS	IIIC omental	3	-	7; 4	0.2013
	Malignant	F	60	Ovary	PS	IA	3	-	6; 4	0.2228
	Malignant	F	61	Ovary	Clear	IC	3	-	5; 4	0.2485
	Malignant	F	44	Ovary	PS	IC	3	-	4; 4	0.2193
	Malignant	F	47	Ovary	PS	IIIC omental and LN	3	-	3; 4	0.3310
	Malignant	F	65	Ovary	PS	IIIC omental and LN	2	-	2; 4	0.2579
	Metastasis	F	75	Ovary	PS	IV	3	-	1; 4	0.2318
	Malignant	F	61	Ovary	PS	IIIC omental	2	-	1; 5	0.2157
	Malignant	F	38	Ovary	PS	IIIC omental	3	-	2; 5	0.2309
	Malignant	F	36	Ovary	PS	IIIC omental	3	-	3; 5	0.1971
	Metastasis	F	60	Fallopian tube	Endometroid	IV	3	-	4; 5	0.9005
	Malignant	F	48	Ovary	Clear	IA	1	-	5; 5	0.2783
	Malignant	F	51	Ovary	Adeno NOS	IIC	3	-	6; 5	0.2500
	Malignant	F	57	Ovary	Adeno NOS	IIIC omental	3	-	7; 5	0.2439
	Malignant	F	48	Ovary	Adeno NOS	IIIC omental	3	-	8; 5	0.2206
	Malignant	F	61	Ovary	Adeno NOS	IIIC LN	3	-	9; 5	0.2222
	Malignant	F	67	Fallopian tube	PS	IIIC omental and LN	3	-	10; 5	0.3543

	Metastasis	F	55	Ovary	Other	IV	3	-	11; 5	0.3299
	Malignant	F	85	Ovary	PS	IIIC omental	3	-	12; 5	0.2293
	Malignant	F	68	Ovary	Endometroid	IIIC omental	3	-	13; 5	0.2194
	Malignant	F	65	Ovary	PS	IIIC omental and LN	3	-	14; 5	0.3811
	Malignant	F	68	Ovary	Clear	IIIC omental	3	-	15; 5	0.2025
	Malignant	F	42	Ovary	Adeno NOS	IA	3	-	16; 5	0.2280
	Malignant	F	58	Ovary	PS	IIIC omental	3	-	17; 5	0.2451
	Malignant	F	56	Ovary	PS	IIIC omental	3	-	18; 5	0.2153
	Malignant	F	51	Ovary	PS	IIIC omental	3	-	19; 5	0.2315
	Malignant	F	69	Ovary	PS	IIIC omental and LN	3	-	20; 5	0.2618
	Malignant	F	46	Ovary	PS	IIIC omental	3	-	21; 5	0.2493
	Malignant	F	78	Ovary	Endometroid	IIIC omental	3	-	22; 5	0.2572
	Malignant	F	43	Ovary	Clear	IIIC omental	3	-	22; 6	0.2121
	Malignant	F	63	Ovary	Clear	IIIC omental	3	-	21; 6	0.2063
	Malignant	F	49	Ovary	PS	IC	3	-	20; 6	0.2035
	Malignant	F	52	Ovary	Endometroid	IIC	3	-	19; 6	0.2200
	Malignant	F	42	Ovary	Mucinous	IA	1	-	18; 6	0.2362
	Malignant	F	85	Ovary	PS	IC	3	-	17; 6	0.2163
	Malignant	F	57	Ovary	PS	IIIC omental	2	-	16; 6	0.2961
	Malignant	F	75	Ovary	PS	IIIC omental	3	-	15; 6	0.2280
	Metastasis	F	72	Ovary	PS	IV	3	-	14; 6	0.2122
	Metastasis	F	76	Ovary	Adeno NOS	IV	3	-	13; 6	0.2565
	Metastasis	F	51	Ovary	PS	IV	3	-	12; 6	0.0000
	Metastasis	F	62	Ovary	Adeno NOS	IV	3	-	11; 6	0.2351
	Malignant	F	76	Ovary	PS	IIIC LN	3	-	10; 6	0.3274
	Malignant	F	73	Ovary	Endometroid	IIIC omental	3	-	9; 6	0.4491
	Malignant	F	41	Ovary	PS	IC	1	-	8; 6	0.2637
	Malignant	F	57	Ovary	PS	IIIC omental and LN	3	-	7; 6	0.2219
	Malignant	F	56	Ovary	PS	IIIC omental	3	-	6; 6	0.2211
	Malignant	F	61	Ovary	PS	IIIC omental	3	-	5; 6	0.2386
	Malignant	F	70	Ovary	PS	IC	3	-	4; 6	0.2564
	Malignant	F	72	Ovary	Endometroid	IIIC omental	3	-	3; 6	0.2909
	Malignant	F	52	Ovary	PS	IIC	1	-	2; 6	0.3149

	Malignant	F	55	Ovary	Other	IIIC omental and LN	2	-	1; 6	0.2290
	Malignant	F	75	Ovary	PS	IIIC omental	3	-	1; 7	0.1899
	Malignant	F	67	Ovary	PS	IIIC omental and LN	3	-	2; 7	0.1870
	Malignant	F	57	Ovary	Endometroid	IIIC omental	3	-	3; 7	0.2149
	Malignant	F	46	Ovary	PS	IIB	1	-	4; 7	0.2236
	Malignant	F	53	Ovary	PS	IIIC omental and LN	3	-	5; 7	0.1932
	Malignant	F	26	Ovary	PS	IIIC omental	2	-	6; 7	0.2316
	Malignant	F	41	Ovary	Clear	IIIC omental	3	-	7; 7	0.2017
	Malignant	F	52	Ovary	Endometroid	IC	3	-	8; 7	0.2409
	Malignant	F	41	Ovary	PS	IIC	1	-	9; 7	0.2543
	Malignant	F	63	Ovary	PS	IIIC omental	3	-	10; 7	0.2286
	Malignant	F	50	Ovary	PS	IIIC omental	3	-	11; 7	0.2377
	Malignant	F	68	Ovary	PS	IIB	3	-	12; 7	0.2056
	Metastasis	F	64	Ovary	Adeno NOS	IV	3	-	13; 7	0.2199
	Malignant	F	60	Ovary	PS	IA	1	-	14; 7	0.3386
	Malignant	F	54	Ovary	PS	IIIC omental	2	-	15; 7	0.0000
	Malignant	F	41	Ovary	Clear	IIIC omental	3	-	16; 7	0.2034
	Malignant	F	55	Ovary	PS	IIIB	3	-	17; 7	0.1659
	Malignant	F	76	Ovary	PS	IIIC omental and LN	3	-	18; 7	0.2617
	Malignant	F	60	Ovary	PS	IIIC omental and LN	3	-	19; 7	0.2582
	Malignant	F	69	Ovary	PS	IIIC omental	3	-	20; 7	0.4322
	Metastasis	F	72	Ovary	PS	IV	3	-	21; 7	0.2375
	Malignant	F	63	Ovary	PS	IA	2	-	22; 7	0.2776
	Malignant	F	49	Ovary	Endometroid	IIA	3	-	22; 8	0.2151
	Malignant	F	52	Ovary	PS	IA	1	-	21; 8	0.2656
	Metastasis	F	76	Ovary	PS	IV	3	-	20; 8	0.1828
	Malignant	F	44	Ovary	PS	IIIC omental	3	-	19; 8	0.2207
	Malignant	F	70	Ovary	PS	IIIC omental	3	-	18; 8	0.1746
	Malignant	F	58	Ovary	Clear	IIIC omental	3	-	17; 8	0.2002
	Malignant	F	76	Ovary	PS	IA	3	-	16; 8	0.2459
	Malignant	F	81	Ovary	PS	IIIC omental	3	-	15; 8	0.1922
	Malignant	F	68	Peritoneum spec parts	PS	IIIC omental	3	-	14; 8	0.2363
	Malignant	F	63	Ovary	PS	IIIC omental	3	-	13; 8	0.2241

	Metastasis	F	54	Ovary	PS	IV	3	-	12; 8	0.2205
	Malignant	F	55	Ovary	PS	IIIC LN	3	-	11; 8	0.2133
	Malignant	F	57	Ovary	Clear	IIC	3	-	10; 8	0.1868
	Malignant	F	64	Ovary	PS	IA	3	-	9; 8	0.2080
	Metastasis	F	76	Ovary	PS	IV	3	-	8; 8	0.2267
	Malignant	F	39	Ovary	PS	IIIC omental and LN	3	-	7; 8	0.2563
	Malignant	F	73	Ovary	PS	IIIC omental	3	-	6; 8	0.2692
	Malignant	F	62	Ovary	Endometroid	IIIC omental	3	-	5; 8	0.2422
	Malignant	F	48	Ovary	PS	IIC	1	-	4; 8	0.2294
	Metastasis	F	63	Ovary	PS	IV	3	-	3; 8	0.2217
	Malignant	F	58	Ovary	PS	IIIB	3	-	2; 8	0.2135
	Metastasis	F	42	Ovary	Clear	IV	3	-	1; 8	0.2354
	Malignant	F	58	Ovary	PS	IA	3	-	1; 9	0.2216
	Malignant	F	49	Ovary	PS	IIIC omental	3	-	2; 9	0.2371
	Metastasis	F	79	Ovary	PS	IV	3	-	3; 9	0.2003
	Metastasis	F	52	Ovary	PS	IV	3	-	4; 9	0.2192
	Malignant	F	78	Ovary	PS	IC	1	-	5; 9	0.4008
	Malignant	F	64	Ovary	PS	IIIC omental	3	-	6; 9	0.2341
	Malignant	F	50	Ovary	PS	IIIA	3	-	7; 9	0.1812
	Malignant	F	46	Ovary	PS	IA	1	-	8; 9	0.2135
	Malignant	F	46	Ovary	Clear	IIIC omental	3	-	9; 9	0.2227
	Malignant	F	60	Ovary	Endometroid	IA	3	-	10; 9	0.3706
	Malignant	F	67	Ovary	Mucinous	IIIA	3	-	11; 9	0.2333
	Malignant	F	67	Ovary	Mucinous	IA	1	-	12; 9	0.2402
	Malignant	F	34	Ovary	PS	IA	1	-	13; 9	0.3433
	Malignant	F	47	Peritoneum nos	PS	IIIC omental and LN	1	-	14; 9	0.2705
	Malignant	F	67	Ovary	PS	IIIC omental and LN	3	-	15; 9	0.2713
	Malignant	F	81	Ovary	PS	IIIC omental	3	-	16; 9	0.2291
	Malignant	F	30	Ovary	PS	IIIC omental	3	-	17; 9	0.2104
	Malignant	F	68	Ovary	PS	IIIC omental	3	-	18; 9	0.2484
	Malignant	F	54	Ovary	Endometroid	IIIC LN	3	-	19; 9	0.2092
	Malignant	F	46	Ovary	Clear	IIA	1	-	20; 9	0.2207
	Malignant	F	32	Ovary	Endometroid	IA	3	-	21; 9	0.2502
	Malignant	F	45	Ovary	PS	IC	1	-	22; 9	0.2483
	Metastasis	F	61	Ovary	PS	IV	3	-	22; 10	0.2799
	Malignant	F	70	Ovary	PS	IIIC omental	3	-	21; 10	0.0000
	Malignant	F	77	Ovary	Endometroid	IIIC omental	3	-	20; 10	0.2652

	Malignant	F	50	Ovary	Clear	IC	1	-	19; 10	0.2230
	Malignant	F	52	Ovary	PS	IIA	3	-	18; 10	0.2347
	Malignant	F	82	Ovary	PS	IA	2	-	17; 10	0.2364
	Malignant	F	79	Ovary	PS	IIIC omental	3	-	16; 10	0.2649
	Malignant	F	57	Ovary	PS	IC	2	-	15; 10	0.2680
	Metastasis	F	49	Ovary	PS	IV	3	-	14; 10	0.1973
	Malignant	F	50	Ovary	PS	IIIC omental	3	-	13; 10	0.2379
	Metastasis	F	56	Ovary	Clear	IV	3	-	12; 10	0.2235
	Malignant	F	68	Ovary	Endometroid	IIC	3	-	11; 10	0.2456
	Malignant	F	51	Ovary	PS	IIIB	2	-	10; 10	0.2154
	Malignant	F	51	Ovary	PS	IIIC omental	3	-	9; 10	0.1947
	Malignant	F	66	Ovary	PS	IIIC omental	3	-	8; 10	0.2308
	Metastasis	F	52	Ovary	PS	IV	3	-	7; 10	0.2500
	Malignant	F	44	Ovary	Adeno NOS	IIIB	3	-	6; 10	0.2363
	Malignant	F	60	Ovary	Endometroid	IIC	3	-	5; 10	0.2046
	Malignant	F	52	Ovary	PS	IIIC omental	1	-	4; 10	0.2228
	Metastasis	F	68	Ovary	PS	IV	3	-	3; 10	0.2324
	Malignant	F	74	Ovary	PS	IIIC LN	3	-	2; 10	0.2211
	Malignant	F	67	Ovary	PS	IIIC omental	3	-	1; 10	0.2434
	Malignant	F	44	Ovary	PS	IIIC omental	3	-	1; 11	0.2135
	Metastasis	F	67	Ovary	PS	IV	3	-	2; 11	0.2272
	Metastasis	F	56	Ovary	PS	IV	3	-	3; 11	0.2347
	Malignant	F	78	Ovary	PS	IIIC omental	3	-	4; 11	0.2097
	Malignant	F	74	Ovary	Endometroid	IIIC omental and LN	3	-	5; 11	0.2307
	Malignant	F	40	Ovary	PS	IIA	2	-	6; 11	0.2156
	Malignant	F	63	Ovary	PS	IIIC omental	3	-	7; 11	0.2186
	Metastasis	F	66	Ovary	Endometroid	IV	1	-	8; 11	0.2006
	Malignant	F	54	Ovary	PS	IA	1	-	9; 11	0.2136
	Malignant	F	65	Ovary	PS	IIIC omental	3	-	10; 11	0.2656
	Malignant	F	80	Ovary	PS	IIIC omental	3	-	11; 11	0.2129
	Malignant	F	84	Ovary	PS	IA	3	-	12; 11	0.2074
	Malignant	F	77	Ovary	PS	IIIC omental	3	-	13; 11	0.2144
	Malignant	F	51	Ovary	PS	IIIC omental and LN	3	-	14; 11	0.2220
	Metastasis	F	42	Ovary	PS	IV	3	-	15; 11	0.1994
	Metastasis	F	54	Ovary	PS	IV	3	-	16; 11	0.2200
	Malignant	F	63	Ovary	Mucinous	IIIC omental	3	-	17; 11	0.2655
	Malignant	F	56	Ovary	PS	IC	3	-	18; 11	0.2443

	Malignant	F	57	Ovary	PS	IIIC omental and LN	3	-	19; 11	0.2751
	Metastasis	F	63	Ovary	Adeno NOS	IV	3	-	20; 11	0.3703
	Malignant	F	45	Ovary	PS	IIA	3	-	21; 11	0.2472
	Metastasis	F	63	Ovary	PS	IV	3	-	22; 11	0.2429
	Metastasis	F	78	Ovary	Clear	IV	3	-	22; 12	0.2303
	Malignant	F	53	Ovary	Other	IIIC omental	3	-	21; 12	0.2569
	Malignant	F	77	Ovary	Clear	IIIC omental	3	-	20; 12	0.2061
	Malignant	F	44	Ovary	PS	IA	3	-	19; 12	0.1914
	Malignant	F	53	Ovary	Clear	IIIC LN	3	-	18; 12	0.2404
	Malignant	F	61	Ovary	PS	IIIC omental	3	-	17; 12	0.2790
	Malignant	F	72	Ovary	PS	IIIC omental	3	-	16; 12	0.3192
	Malignant	F	69	Ovary	PS	IIIC omental	3	-	15; 12	0.2090
	Malignant	F	34	Ovary	PS	IIIC omental and LN	3	-	14; 12	0.2616
	Malignant	F	58	Peritoneum spec parts	PS	IIIC omental	3	-	13; 12	0.2513
	Malignant	F	60	Ovary	PS	IIC	3	-	12; 12	0.2579
	Malignant	F	74	Ovary	PS	IIIC omental	2	-	11; 12	0.2198
	Malignant	F	53	Ovary	PS	IIIC omental	3	-	10; 12	0.2404
	Malignant	F	78	Ovary	Adeno NOS	IIIC omental	3	-	9; 12	0.2206
	Malignant	F	51	Ovary	PS	IIA	3	-	8; 12	0.2380
	Malignant	F	84	Ovary	Clear	IIIC omental	3	-	7; 12	0.2181
	Malignant	F	54	Ovary	PS	IC	1	-	6; 12	0.2197
	Malignant	F	71	Ovary	PS	IIIC omental	3	-	5; 12	0.2086
	Malignant	F	55	Ovary	Clear	IIIC omental	3	-	4; 12	0.2344
	Malignant	F	59	Ovary	PS	IIA	3	-	3; 12	0.2375
	Malignant	F	51	Ovary	PS	IIIC omental	3	-	2; 12	0.2484
	Malignant	F	54	Ovary	PS	IA	3	-	1; 12	0.2611
	Metastasis	F	62	Ovary	Adeno NOS	IV	3	-	1; 13	0.2952
	Malignant	F	62	Ovary	PS	IIC	3	-	2; 13	0.2175
	Malignant	F	65	Ovary	PS	IIIC omental	3	-	3; 13	0.0000
	Malignant	F	56	Ovary	Other	IIIC omental and LN	3	-	4; 13	0.2481
	Malignant	F	77	Ovary	PS	IIC	3	-	5; 13	0.2104
	Malignant	F	50	Ovary	Endometroid	IIIC omental	3	-	6; 13	0.2021
	Malignant	F	57	Ovary	PS	IIIB	1	-	7; 13	0.2040
	Malignant	F	83	Ovary	PS	IIIB	3	-	8; 13	0.2623
	Malignant	F	62	Ovary	PS	IIIB	3	-	9; 13	0.2255

	Malignant	F	64	Ovary	Mucinous	IIA	3	-	10; 13	0.2224
	Malignant	F	12	Ovary	PS	IA	2	-	11; 13	0.2014
	Malignant	F	49	Fallopian tube	PS	IC	3	-	12; 13	0.2273
	Malignant	F	67	Ovary	PS	IIIC omental	3	-	13; 13	0.2316
	Metastasis	F	50	Ovary	PS	IV	3	-	14; 13	0.3705
	Malignant	F	39	Ovary	PS	IIIC omental	3	-	15; 13	0.2776
	Malignant	F	25	Ovary	PS	IIIC omental	3	-	16; 13	0.2054
	Malignant	F	51	Ovary	PS	IIIC omental	3	-	17; 13	0.2823
	Malignant	F	56	Ovary	Endometroid	IA	3	-	18; 13	0.2602
	Malignant	F	48	Ovary	PS	IIIC omental	1	-	19; 13	0.2140
	Malignant	F	59	Ovary	Endometroid	IIIC omental	3	-	20; 13	0.2800
	Malignant	F	79	Ovary	Clear	IC	3	-	21 13	0.2560
	Malignant	F	47	Ovary	PS	IIIC LN	3	-	22; 13	0.0000
	Malignant	F	76	Ovary	PS	IIIC omental and LN	3	-	22; 14	0.2589
	Malignant	F	84	Ovary	PS	IIIC omental	3	-	21; 14	0.2358
	Malignant	F	69	Ovary	PS	IIIC omental	3	-	20; 14	0.2054
	Malignant	F	64	Ovary	PS	IIIC omental	3	-	19; 14	0.2298
	Malignant	F	67	Ovary	PS	IIIC omental	3	-	18; 14	0.2698
	Metastasis	F	80	Ovary	PS	IV	3	-	17; 14	0.2117
	Malignant	F	70	Ovary	Clear	IIIC omental	3	-	16; 14	0.2401
	Malignant	F	56	Ovary	Clear	IIIC omental	3	-	15; 14	0.2631
	Malignant	F	73	Ovary	PS	IC	3	-	14; 14	0.2107
	Metastasis	F	57	Ovary	PS	IV	3	-	13; 14	0.2426
	Malignant	F	31	Ovary	PS	IIIC omental	3	-	12; 14	0.1923
	Metastasis	F	63	Ovary	PS	IV	3	-	11; 14	0.2410
	Malignant	F	65	Ovary	Clear	IIIC omental and LN	3	-	10; 14	0.1960
	Metastasis	F	73	Ovary	PS	IV	3	-	9; 14	0.2180
	Malignant	F	61	Ovary	Adeno NOS	IIIC omental	3	-	8; 14	0.2366
	Malignant	F	50	Ovary	PS	IIIC omental and LN	3	-	7; 14	0.1868
	Malignant	F	69	Ovary	Mucinous	IC	2	-	6; 14	0.2163
	Malignant	F	46	Ovary	PS	IA	3	-	5; 14	0.2284
	Malignant	F	66	Ovary	PS	IIIC omental	3	-	4; 14	0.2581
	Metastasis	F	59	Ovary	PS	IV	3	-	3; 14	0.2593
	Malignant	F	77	Ovary	PS	IIIB	3	-	2; 14	0.2201
	Malignant	F	53	Ovary	Endometroid	IIIC omental	3	-	1; 14	0.2220

	Malignant	F	52	Ovary	Endometroid	IIC	1	-	1; 15	0.4493
	Malignant	F	64	Ovary	PS	IA	1	-	2; 15	0.3368
	Metastasis	F	58	Ovary	Clear	IV	3	-	3; 15	0.0000
	Malignant	F	32	Ovary	PS	IIIC omental and LN	3	-	4; 15	0.2154
	Malignant	F	40	Ovary	Endometroid	IA	1	-	5; 15	0.2707
	Malignant	F	65	Ovary	PS	IA	2	-	6; 15	0.2209
	Malignant	F	75	Ovary	Mucinous	IIIC omental and LN	3	-	7; 15	0.2360
	Malignant	F	62	Ovary	PS	IIC	3	-	8; 15	0.2292
	Malignant	F	68	Ovary	PS	IIB	2	-	9; 15	0.2484
	Malignant	F	70	Ovary	PS	IIIC omental	3	-	10; 15	0.2334
	Malignant	F	55	Ovary	PS	IIIC omental	3	-	11; 15	0.2077
	Malignant	F	74	Fallopian tube	PS	IIIC omental	3	-	12; 15	0.2140
	Malignant	F	44	Ovary	PS	IIIC omental	3	-	13; 15	0.2066
	Malignant	F	46	Ovary	PS	IIIC omental and LN	3	-	14; 15	0.2434
	Malignant	F	62	Ovary	PS	IIIC omental	3	-	15; 15	0.2295
	Malignant	F	75	Ovary	Endometroid	IIA	3	-	16; 15	0.2575
	Malignant	F	56	Ovary	PS	IC	3	-	17; 15	0.2410
	Malignant	F	82	Ovary	PS	IIIC omental	3	-	18; 15	0.2563
	Malignant	F	62	Ovary	PS	IIIC omental	3	-	19; 15	0.2595
	Malignant	F	61	Ovary	PS	IIIC omental	3	-	20; 15	0.2041
	Malignant	F	77	Ovary	Mucinous	IIIC omental and LN	3	-	21; 15	0.2414
	Malignant	F	51	Ovary	PS	IA	1	-	22; 15	0.3092
	Malignant	F	66	Ovary	PS	IIIC omental	3	-	22; 16	0.2719
	Malignant	F	44	Ovary	PS	IIC	3	-	21; 16	0.2582
	Malignant	F	75	Ovary	Endometroid	IIC	3	-	20; 16	0.3007
	Malignant	F	34	Ovary	PS	IIIC LN	1	-	19; 16	0.2567
	Malignant	F	47	Ovary	Endometroid	IIC	3	-	18; 16	0.6089
	Malignant	F	64	Ovary	PS	IIC	3	-	17; 16	0.2398
	Malignant	F	66	Ovary	PS	IIIC omental and LN	3	-	16; 16	0.2386
	Malignant	F	50	Ovary	PS	IIIC LN	3	-	15; 16	0.2491
	Malignant	F	61	Ovary	PS	IIIB	3	-	14; 16	0.2230
	Metastasis	F	82	Ovary	PS	IV	3	-	13; 16	0.2172
	Malignant	F	40	Ovary	PS	IA	3	-	12; 16	0.2483
	Malignant	F	77	Ovary	PS	IIIC omental and LN	3	-	11; 16	0.2448

	Malignant	F	67	Ovary	Mucinous	IIIC omental	3	-	10; 16	0.2681
	Malignant	F	38	Ovary	PS	IC	1	-	9; 16	0.2435
	Malignant	F	78	Ovary	PS	IA	3	-	8; 16	0.2605
	Malignant	F	60	Ovary		IIIC omental	3	-	7; 16	0.2389
Testis tissues (catalog no. TE803)										
	**Tissue type**	**Sex**	**Age**	**Organ**	**Pathology**	**Stage**	**Grade**	**TNM**	**Position**	**PLK5 expression**
	malignant	M	59	Testis	Seminoma	I	-	T4N0M0	A1	0.0000
	malignant	M	52	Testis	Seminoma with necrosis	I	-	T2N0M0	A2	0.3470
	malignant	M	31	Testis	Seminoma (fibrous tissue, blood vessel and necrotic tissue)	I	-	T1N0M0	A3	0.3373
	malignant	M	38	Testis	Seminoma	I	-	T1N0M0	A4	0.0000
	malignant	M	45	Testis	Seminoma	I	-	T1N0M0	A5	0.0000
	malignant	M	52	Testis	Seminoma (sparse)	I	-	T2N0M0	A6	0.0000
	malignant	M	32	Testis	Seminoma	I	-	T1N0M0	A7	0.0000
	malignant	M	28	Testis	Seminoma	I	-	T2N0M0	A8	0.0000
	malignant	M	36	Testis	Seminoma	I	-	T2N0M0	A9	0.0000
	malignant	M	70	Testis	Seminoma	I	-	T1N0M0	A10	0.0000
	malignant	M	40	Testis	Seminoma	I	-	T2N0M0	B1	0.0000
	malignant	M	30	Testis	Seminoma (sparse)	I	-	T1N0M0	B2	0.0000
	malignant	M	38	Testis	Seminoma	I	-	T1N0M0	B3	0.0000
	malignant	M	42	Testis	Seminoma	I	-	T2N0M0	B4	0.0000
	malignant	M	44	Testis	Seminoma	I	-	T1N0M0	B5	0.0000
	malignant	M	35	Testis	Seminoma	I	-	T4NOMO	B6	0.0000
	malignant	M	34	Testis	Seminoma	I	-	T1N0M0	B7	0.0000
	malignant	M	56	Testis	Seminoma	I	-	T2N0M0	B8	0.0000
	malignant	M	50	Testis	Seminoma (sparse) with necrosis	III	-	T2N0M1	B9	0.2017
	malignant	M	39	Testis	Seminoma	I	-	T2N0M0	B10	0.0000
	malignant	M	29	Testis	Seminoma	I	-	T2N0M0	C1	0.0000
	malignant	M	34	Testis	Seminoma	I	-	T1N0M0	C2	0.0000
	malignant	M	30	Testis	Seminoma	I	-	T2N0M0	C3	0.0000
	malignant	M	44	Testis	Seminoma	I	-	T2N0M0	C4	0.0000
	malignant	M	48	Testis	Seminoma	I	-	T1N0M0	C5	0.0000
	malignant	M	33	Testis	Seminoma	I	-	T1N0M0	C6	0.2698
	malignant	M	25	Testis	Embryonal carcinoma	II	-	T4N1M0	C7	0.3522
	malignant	M	38	Testis	Embryonal carcinoma	I	-	T1N0M0	C8	0.3050
	malignant	M	28	Testis	Embryonal carcinoma	I	-	T1N0M0	C9	0.2335
	malignant	M	43	Testis	Embryonal carcinoma	I	-	T2N0M0	C10	0.0000
	malignant	M	36	Testis	Embryonal carcinoma	I	-	T1N0M0	D1	0.3397
	malignant	M	30	Testis	Embryonal carcinoma	I	-	T2N0M0	D2	0.3590
	malignant	M	32	Testis	Embryonal carcinoma (contorted seminiferous tubules sparse)	I	-	T2N0M0	D3	0.3009
	malignant	M	23	Testis	Embryonal carcinoma	I	-	T1N0M0	D4	0.2890
	malignant	M	21	Testis	Embryonal carcinoma	I	-	T2N0M0	D5	0.2677
	malignant	M	26	Testis	Embryonal carcinoma (sparse)	I	-	T1N0M0	D6	0.2510

	malignant	M	30	Testis	Embryonal carcinoma (atrophy)	I	-	T1N0M0	D7	0.4933
	malignant	M	18	Testis	Embryonal carcinoma	I	-	T3N0M0	D8	0.3313
	malignant	M	2	Testis	Yolk sac tumor	I	-	T1N0M0	D9	0.2135
	malignant	M	52	Testis	Yolk sac tumor	I	-	T1N0M0	D10	0.0000
	malignant	M	8 Mon.	Testis	Yolk sac tumor	I	-	T2N0M0	E1	0.2533
	malignant	M	2	Testis	Yolk sac tumor	I	-	T1N0M0	E2	0.2612
	malignant	M	30	Testis	Yolk sac tumor	I	-	T1N0M0	E3	0.2826
	malignant	M	32	Testis	Yolk sac tumor	I	-	T1N0M0	E4	0.2636
	malignant	M	23	Testis	Yolk sac tumor	I	-	T1N0M0	E5	0.2819
	malignant	M	2	Testis	Yolk sac tumor	I	-	T1N0M0	E6	0.3210
	malignant	M	15	Testis	Yolk sac tumor	I	-	T1N0M0	E7	0.3438
	malignant	M	21	Testis	Yolk sac tumor	I	-	T1N0M0	E8	0.3566
	malignant	M	35	Testis	Immature teratoma	I	-	T1N0M0	E9	0.2356
	malignant	M	32	Testis	Immature teratoma	I	-	T1N0M0	E10	0.0000
	malignant	M	44	Testis	Immature teratoma	I	-	T2N0M0	F1	0.2993
	benign	M	48	Testis	Mature teratoma	-	-	-	F2	0.3216
	benign	M	39	Testis	Mature teratoma	-	-	-	F3	0.3591
	benign	M	31	Testis	Mature teratoma	-	-	-	F4	0.3352
	benign	M	13	Testis	Mature teratoma	-	-	-	F5	0.4615
	TB	M	31	Testis	Tuberculosis	-	-	-	F6	0.2921
	TB	M	27	Testis	Tuberculosis	-	-	-	F7	0.2923
	TB	M	40	Testis	Tuberculosis	-	-	-	F8	0.0000
	TB	M	68	Testis	Tuberculosis	-	-	-	F9	0.0000
	atrophy	M	77	Testis	Atrophy	-	-	-	F10	0.0000
	atrophy	M	87	Testis	Atrophy	-	-	-	G1	0.0000
	atrophy	M	61	Testis	Atrophy	-	-	-	G2	0.0000
	atrophy	M	83	Testis	Atrophy	-	-	-	G3	0.0000
	atrophy	M	52	Testis	Atrophy	-	-	-	G4	0.0000
	atrophy	M	68	Testis	Atrophy	-	-	-	G5	0.0000
	NAT	M	65	Testis	Cancer adjacent normal testicular tissue	-	-	-	G6	0.2917
	NAT	M	74	Testis	Cancer adjacent normal testicular tissue	-	-	-	G7	0.0000
	NAT	M	61	Testis	Cancer adjacent normal testicular tissue	-	-	-	G8	0.0000
	NAT	M	74	Testis	Cancer adjacent normal testicular tissue	-	-	-	G9	0.3682
	NAT	M	69	Testis	Cancer adjacent normal testicular tissue	-	-	-	G10	0.0000
	NAT	M	70	Testis	Cancer adjacent normal testicular tissue	-	-	-	H1	0.0000
	NAT	M	46	Testis	Cancer adjacent normal testicular tissue	-	-	-	H2	0.2574
	NAT	M	54	Testis	Cancer adjacent normal testicular tissue	-	-	-	H3	0.0000
	NAT	M	21	Testis	Cancer adjacent normal testicular tissue	-	-	-	H4	0.0000
	NAT	M	39	Testis	Cancer adjacent normal testicular tissue	-	-	-	H5	0.0000
	normal	M	45	Testis	Normal testicular tissue	-	-	-	H6	0.0000
	normal	M	30	Testis	Normal testicular tissue	-	-	-	H7	0.0000

	normal	M	45	Testis	Normal testicular tissue	-	-	-	H8	0.0000
	normal	M	45	Testis	Normal testicular tissue	-	-	-	H9	0.0000
	normal	M	46	Testis	Normal testicular tissue	-	-	-	H10	0.0000
